# In Situ Structural Evolution and Activity Descriptor of Atomically Dispersed Catalysts During Nitrate Electroreduction

**DOI:** 10.1002/advs.202510282

**Published:** 2025-08-22

**Authors:** Daniel S. Braga, Angus Pedersen, Mohd Riyaz, Jesús Barrio, Alexander Bagger, Itamar T. Neckel, Thiago M. Mariano, Manuel E. G. Winkler, Ifan E. L. Stephens, Maria‐Magdalena Titirici, Raphael Nagao

**Affiliations:** ^1^ Institute of Chemistry University of Campinas Campinas SP 13083‐862 Brazil; ^2^ Department of Chemical Engineering Imperial College London London SW7 2AZ United Kingdom; ^3^ Division 3.6 Electrochemical Energy Materials Bundesanstalt für Materialprüfung und ‐forschung (BAM) Unter den Eichen 44‐46 12203 Berlin Germany; ^4^ Department of Physics Technical University of Denmark Kongens Lyngby 2800 Denmark; ^5^ Brazilian Synchrotron Light Laboratory Brazilian Center for Research in Energy and Materials Campinas SP 13083‐100 Brazil; ^6^ Center for Innovation on New Energies University of Campinas Campinas SP 13083‐084 Brazil; ^7^ Department of Materials Imperial College London London SW7 2AZ United Kingdom; ^8^ Advanced Institute for Materials Research (WPI‐AIMR) Tohoku University 2‐1‐1 Katahira, Aobaku Sendai Miyagi 980‐8577 Japan

**Keywords:** green ammonia, single‐atom catalysts, structure‐activity, synchrotron X‐ray fluorescence

## Abstract

Single‐Atom Catalysts (SAC) have emerged as a promising class of materials for various catalytic applications, including the electrochemical nitrate reduction reaction (eNO_3_RR) and consequently ammonia production. While the efficiency and selectivity of these materials have been extensively highlighted for the eNO_3_RR, the in situ evolution to their structure and composition during electrocatalysis is largely unexplored and lacks catalyst design principles. To solve this, we investigated a series of high utilization metal‐nitrogen‐carbon (MNC) SACs (M = Cr, Fe, Co, Ni, and Cu) for eNO_3_RR. Except for CuNC, which selectively produced nitrite, all catalysts exhibited Faradaic efficiencies (FE) for ammonia exceeding 50%. NiNC demonstrated the highest performance (FE of 78.0 ± 2.9% at −0.4 V versus reversible hydrogen electrode (RHE) at pH 13 and maximum ammonia production rate of 615.7 ± 176.5 µmol·h^−1^·${\mathrm{cm}}_{\mathrm{geo}}^{-2}$, corresponding to an energy efficiency of 15.1 ± 1.4% at −0.6 V_RHE_), followed by CoNC. In situ Synchrotron X‐ray fluorescence (SXRF) mapping at various cathodic potentials (from open circuit potential to 0.0 V_RHE_ and then −0.6 V_RHE_ at 100 mV steps) revealed significant mobility of Ni within the carbon matrix, leading to the formation of metallic clusters from 0.0 V_RHE_. Similar in situ metal clustering is observed for CoNC. Structure‐activity plots are generated from both MNC literature and results obtained here, finding a clear trend between OH binding energy and turnover frequency, with the high activity of NiNC and CoNC in this work explained by their stronger OH binding in the metallic structure compared to their SAC coordination. This work therefore, reveals the structure‐activity‐stability of MNCs for eNO_3_RR and provides a simple descriptor for identifying highly active eNO_3_RR catalysts and their in situ structural evolution.

## Graphical abstract

The in situ evolution of single‐atom catalysts to metallic nanoclusters during nitrate electroreduction is demonstrated. A direct correlation between the OH binding energy on the metallic clusters and ammonia production rate is established as a key activity descriptor, explaining the superior performance of Ni and Co catalysts.

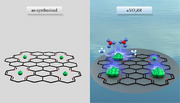

## 1 Introduction

Ammonia (NH_3_) production by the Haber‐Bosch process revolutionized agriculture by enabling global food production to sustain unprecedented population growth—from 1.6 billion in 1900 to over 8 billion in 2024—through synthetic fertilizers.^[^ ^1^, ^2^ ^]^ However, despite 80% of ammonia being used in fertilizers, less than half of the applied nitrogen is absorbed by crops.^[^ ^3^, ^4^ ^]^ The excess leaches into the environment, contaminating the soil, water, and air,^[^ ^2^, ^5^ ^]^ which are converted to nitrate (NO_3_^−^) by nitrifying bacteria.^[^ ^6^ ^]^ Nitrate is highly soluble and persistent, eventually making its way into groundwater and bodies of water, where it poses serious health risks. In drinking water, high nitrate levels can cause methemoglobinemia, or “blue baby syndrome”, in infants, which hinders the blood's ability to carry oxygen.^[^ ^7^, ^8^ ^]^ While adults can tolerate higher nitrate levels, concerns persist regarding potential links to cancer and reproductive issues.^[^ ^8^ ^]^ Beyond health risks, nitrate pollution imposes significant economic burdens, including expensive water treatment, environmental remediation, and agricultural productivity losses.^[^ ^2^ ^]^ While unlikely to replace the sheer industrial scale of the Haber‐Bosch process, eNO_3_RR presents a compelling strategy for simultaneously remediating this widespread pollution and valorizing waste NO_3_^−^ into NH_3_.^[^ ^9^ ^]^

In recent years, the search for more sustainable methods to produce ammonia and mitigate nitrate pollution has intensified. Nitrate electroreduction (eNO_3_RR) has emerged as a promising solution, offering the potential to produce ammonia while simultaneously addressing environmental contamination.^[^ ^10^ ^]^ This technology is gaining attention not only for its ability to generate NH_3_, but also for its applications in nuclear waste management and pollution control.^[^ ^11^, ^12^, ^13^ ^]^ As the world faces growing challenges in food security and environmental sustainability, selective ammonia catalysts and the eNO_3_RR emerge as innovative technologies with the potential to enhance sustainable agricultural practices and reduce environmental impacts.^[^ ^12^, ^14^ ^]^

eNO_3_RR can generate various nitrogenous products, including nitrite, nitric oxide, nitrous oxide, nitrogen, hydroxylamine, and ammonia.^[^ ^15^, ^16^, ^17^, ^18^, ^19^, ^20^ ^]^ In alkaline media (pH 13) the reversible potential for nitrate reduction to nitrite and to ammonia is the following:^[^ ^20^, ^21^, ^22^ ^]^

(1)

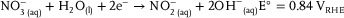



(2)

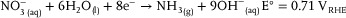

eNO_3_RR studies have been conducted using a range of catalysts, such as metals electrodes,^[^ ^23^, ^24^, ^25^, ^26^ ^]^ metal oxides,^[^ ^16^, ^27^, ^28^, ^29^, ^30^ ^]^ metal‐organic frameworks (MOFs)^[^ ^31^, ^32^, ^33^, ^34^, ^35^ ^]^ and metal phosphides.^[^ ^36^, ^37^, ^38^, ^39^ ^]^ Central to this innovation, single‐atom catalysts (SACs) rise as a cheap, selective, and resource‐efficient alternative catalyst to convert NO_3_^−^ to NH_3_.^[^ ^21^ ^]^ SACs consist of atomically dispersed metal atoms bonded to a support, such as N‐doped carbon (MNC, where M = metal). Their unique structure can, in theory, maximize atomic utilization, and their activity can be tuned based on the doped metal and coordination environment, offering a path forward for both ammonia synthesis and nitrate reduction.^[^ ^40^, ^41^ ^]^

Murphy et al.^[^ ^21^ ^]^ recently showed that SAC FeNC combined with Fe_2_O_3_ can achieve industrial current densities; however, a comprehensive understanding of how catalyst structure influences activity and selectivity is lacking. Additionally, Xi et al. reported mmol production of ammonia based on SAC NiNC combined with Ni nanoparticles on carbon nanotubes.^[^ ^42^ ^]^ This suggests certain cooperativity between MNC and metal clusters under certain conditions.^[^ ^43^ ^]^ Detailed structure‐activity relationships for SACs and metal clusters in eNO_3_RR are limited, hindering rational catalyst design. Murphy et al.^[^ ^14^ ^]^ investigated activity and selectivity across a range of MNC SACs, highlighting that at neutral pH, Fe and Cr exhibited nearly 100% faradaic efficiency (FE); and Wu et al.^[^ ^44^ ^]^ demonstrated that FeNC SAC achieved an ammonia FE of approximately 75% under neutral pH conditions and in pH 13. However, the underlying electronic and structural reasons remain to be fully elucidated. Another crucial gap is the definitive identification of active sites. Li et al. probed the number of active sites in their FeNC SAC and nanoparticle Fe on NC, finding approximately an order of magnitude higher turnover frequency to NH_3_ (TOF_NH3_) for the single‐atom FeNC.^[^ ^45^ ^]^ While these studies have unequivocally confirmed the ex situ single‐atom nature of the active sites using advanced characterization techniques, further efforts are needed to elucidate the dynamic structural evolution of these single sites under reaction conditions. This is crucial for a deeper understanding of the reaction mechanism and for optimizing catalyst design.

In addition to Fe‐based SACs, Cu‐based SACs have also been studied in great depth. Zhu et al.^[^ ^46^ ^]^ demonstrated that CuNC SAC embedded in carbon nanosheets exhibit enhanced activity and stability for nitrate electroreduction compared to bulk copper materials, while also significantly reducing nitrite accumulation. This superior performance was attributed to the unique electronic structure and maximized atom utilization of single‐atom copper. However, subsequent research by Yang et al.^[^ ^47^ ^]^ revealed a dynamic restructuring of Cu SACs during nitrate electroreduction. *Operando* studies showed that the applied potential drives the transformation of isolated Cu atoms into Cu nanoparticles, concurrent with an increase in the ammonia production rate.^[^ ^47^ ^]^ This finding suggests that, contrary to initial assumptions, Cu nanoparticles are the primary active species for ammonia formation. Density functional theory (DFT) calculations further corroborated this, revealing favorable thermodynamics for nitrate‐to‐ammonia conversion on Cu clusters compared to Cu SACs.^[^ ^47^ ^]^ The dynamic interconversion between Cu single‐atoms and nanoparticles, driven by potential and environmental conditions, underscores the complex nature of SACs and highlights the necessity of in situ and *operando* studies to identify their true active sites during nitrate reduction. Together, these insights underline the importance of understanding both static and dynamic aspects of single‐atom catalyst behavior to optimize their design for nitrate reduction reactions.

Additional MNCs may also suffer from limited stability (e.g. demetallation, protonation, carbon oxidation) under harsh reducing or oxidizing electrochemical conditions.^[^ ^48^ ^]^ From the electrochemical CO_2_ reduction reaction (CO_2_RR) literature, Cuenya and coworkers discovered from *operando* X‐ray absorption studies on MNC (M = Cu, Ni, Zn) under −0.55 and −1.15 V_RHE_ that all MNC, except NiNC, underwent partial or complete transformations from single‐atoms to metal particles phases (pure metal, clusters or oxides).^[^ ^49^ ^]^ Additionally, CoNC, ZnNC, and CuNC showed some reversibility back to single‐atoms. A limitation of the work by Cuenya and coworkers was their preparation of MNCs, which involved a 1000 °C pyrolyzed zeolitic imidazolate framework‐8 (ZIF‐8) being impregnated with an active metal salt and subsequently pyrolyzed again (700 °C). This resulted in their as‐synthesised FeNC and SnNC being purely composed of γ‐Fe_2_O_3_ and SnO_2_, respectively. Additionally, from the O_2_ reduction reaction literature, it has been shown that the electrochemical active site utilisation of ZIF‐8‐derived FeNC (without porosity modification) is < 10%,^[^ ^50^ ^]^ based on single‐atom active probing via in situ nitrite stripping (assuming five electron process). Therefore, *operando* bulk characterisation techniques, such as XAS,^[^ ^51^ ^]^ may not be providing a representative picture of the electrochemically accessible active sites, especially if high catalyst loadings are used.^[^ ^52^ ^]^ Combining different *operando* techniques can provide insights at electrocatalyst interfaces;^[^ ^53^ ^]^ however, such studies are highly labour and resource intensive. Alternatively, to overcome active site accessibility limitations, we previously reported a Mg‐based decoupled pyrolysis and subsequent low temperature (90 °C) active site loading approach to produce purely single‐atom MNC powders (M = Fe, Ni) with electrochemical active site utilisation > 50% (based on five electron nitrite stripping).^[^ ^54^, ^55^ ^]^ This approach was based on the method reported by Fellinger and coworkers, where Mg acts both as an active site template and a porogen to generate high specific surface area and porous MgNC.^[^ ^56^ ^]^ The active metal can then be (trans)metalated at a low temperature (80–90 °C), to produce MNC SACs.^[^ ^56^ ^]^ The synthesised Mg‐templated MNCs can therefore serve as model SACs for understanding structure‐selectivity‐stability,^[^ ^57^ ^]^ due to the equivalent chemical and morphological aspects of the MNC, aside from doped metal. For instance, it was recently found from *online* ICP‐MS on our Mg‐templated FeNC under acidic O_2_ reduction that high rates of Fe dissolution occur at open circuit potential (OCP ≈0.9 V_RHE_), leading to formation of Fe_2_O_3_ at elevated current densities (≥ −15 mA·cm^−2^), as expected from the Fe Pourbaix diagram, due to increased local pH.^[^ ^58^ ^]^

The current work focuses on the study of five atomically dispersed metals (Cr, Fe, Co, Ni, and Cu) supported on a carbon‐nitrogen matrix (derived from 2,4,6‐triaminopyrimidine) based on the Mg‐templating approach. We found that NiNC, under conditions of 0.1 mol·L^−1^ NaOH (pH 13) and 0.5 mol·L^−1^ NaNO_3_, deposited on carbon cloth, produced the highest partial ammonia current density and ammonia yield rate (−132 mA·${\mathrm{cm}}_{\mathrm{geo}}^{-2}$ and ≈615 µmol·h^−1^·${\mathrm{cm}}_{\mathrm{geo}}^{-2}$, respectively) at a potential of −0.6 V_RHE_, with a FE of ≈80% at −0.4 V_RHE_. A trend was observed among the transition metals, starting with CrNC, showing increasing ammonia production up to NiNC. However, this trend was broken with CuNC, which produced more nitrite than ammonia, but generating ammonia already at −0.1 V_RHE_. The stability of the NiNC and CoNC catalysts was investigated using synchrotron X‐ray fluorescence (SXRF) mapping, which provided detailed insights into their structural changes under reducing conditions. The analysis revealed that both NiNC and CoNC tended to form metal clusters during the application of reducing potentials. In addition, results from density functional theory (DFT) calculations corroborate these experimental findings, suggesting that the high ammonia production rates can only be achievable by the clustered metal structures.

## 2 Results and Discussion

### 2.1 Synthesis and Characterization

**Figure 1:**
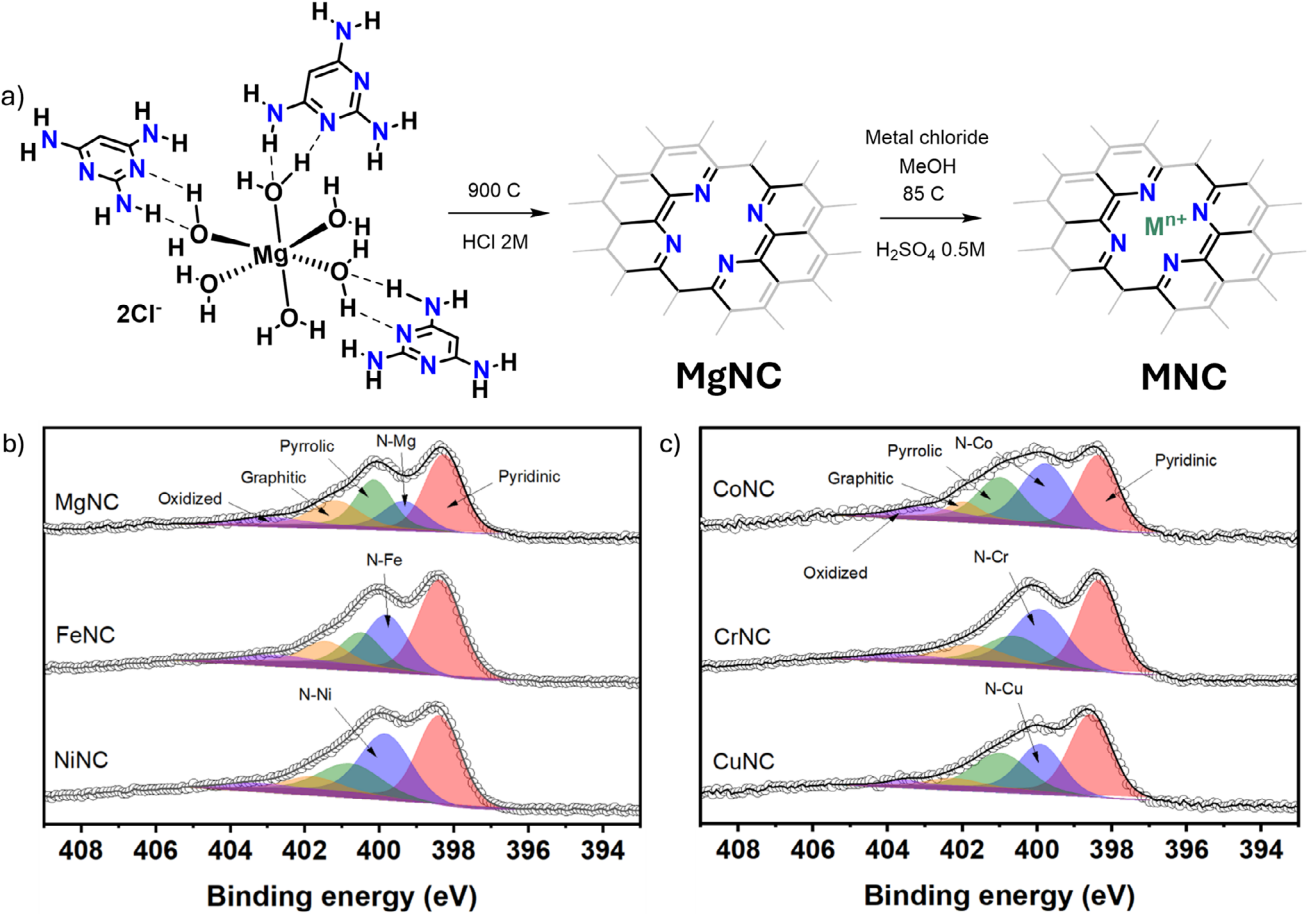
Schematic representation of the synthesis protocol employed, based on the low‐temperature Mg exchange (a). N1s XPS spectra of as prepared metal‐nitrogen‐carbon catalysts (b and c).

Single‐atoms within nitrogen‐doped carbon where prepared as reported previously by low‐temperature metal coordination in a highly porous matrix derived from the Mg‐templated pyrolysis of 2,4,6‐Triaminopyrimidine (TAP).^[^ ^54^ ^]^ MgCl_2_^.^6H_2_O acts as porogen creating a high micro and mesopore volume through MgO generated during high temperature pyrolysis. Mg^2+^ also acts as an active site template and as a Lewis acid interacts efficiently with amine‐based molecules (such as TAP, **Figure** **1**a).^[^ ^56^, ^59^ ^]^ Therefore, after pyrolysis at 900 °C, a nitrogen doped carbon is obtained with a specific surface area of > 3000 m^2^·g^−1^.^[^ ^56^, ^59^ ^]^ The low temperature coordination of metals in Mg‐templated nitrogen‐carbons allows the formation of single metallic sites via magnesium exchange, and through the coordination in free nitrogen functionalities, avoiding their aggregation at high temperatures and resulting in the unequivocal formation of SACs.^[^ ^60^ ^]^ ICP‐MS on the MNCs confirms the presence of the doped active metals within the materials in amounts ranging 0.77–1.99 wt% (Figure S1a, Supporting Information), corresponding to 0.18–0.38 at% (Figure S1b, Supporting Information). ICP‐MS data were converted from wt% to at% according to Equation S1 (Supporting Information).

X‐ray diffraction (XRD) measurements on the range of MNC (Figure S2, Supporting Information) show an absence of sharp defined peaks corresponding to metal nanoparticles as well as limited graphite peaks, which are indicative of atomically thin carbon planes with limited carbon stacking. Additionally, X‐ray photoelectron spectroscopy (XPS) confirms the presence of metallic moieties in the surface (Figure S3, Supporting Information) and suggests the presence of nitrogen‐coordinated metals in the prepared materials (Figure 1b,c). This contribution can be observed at a binding energy 399.75 ± 0.22 eV, which comprises between 15 (in the case of the reference MgNC) to 35 at% (Figure S4, Supporting Information). Besides N‐Metal, contributions corresponding to pyridinic (398.39 ± 0.1 eV), pyrrolic (400.66 ± 0.32 eV), graphitic (401.74 ± 0.39 eV) and oxidized nitrogen (403.30 ± 0.35 eV) are also observed (Table S1, Supporting Information), as well as very similar features in the C1s spectra of all the materials (Figure S5, Supporting Information).

**Figure 2:**
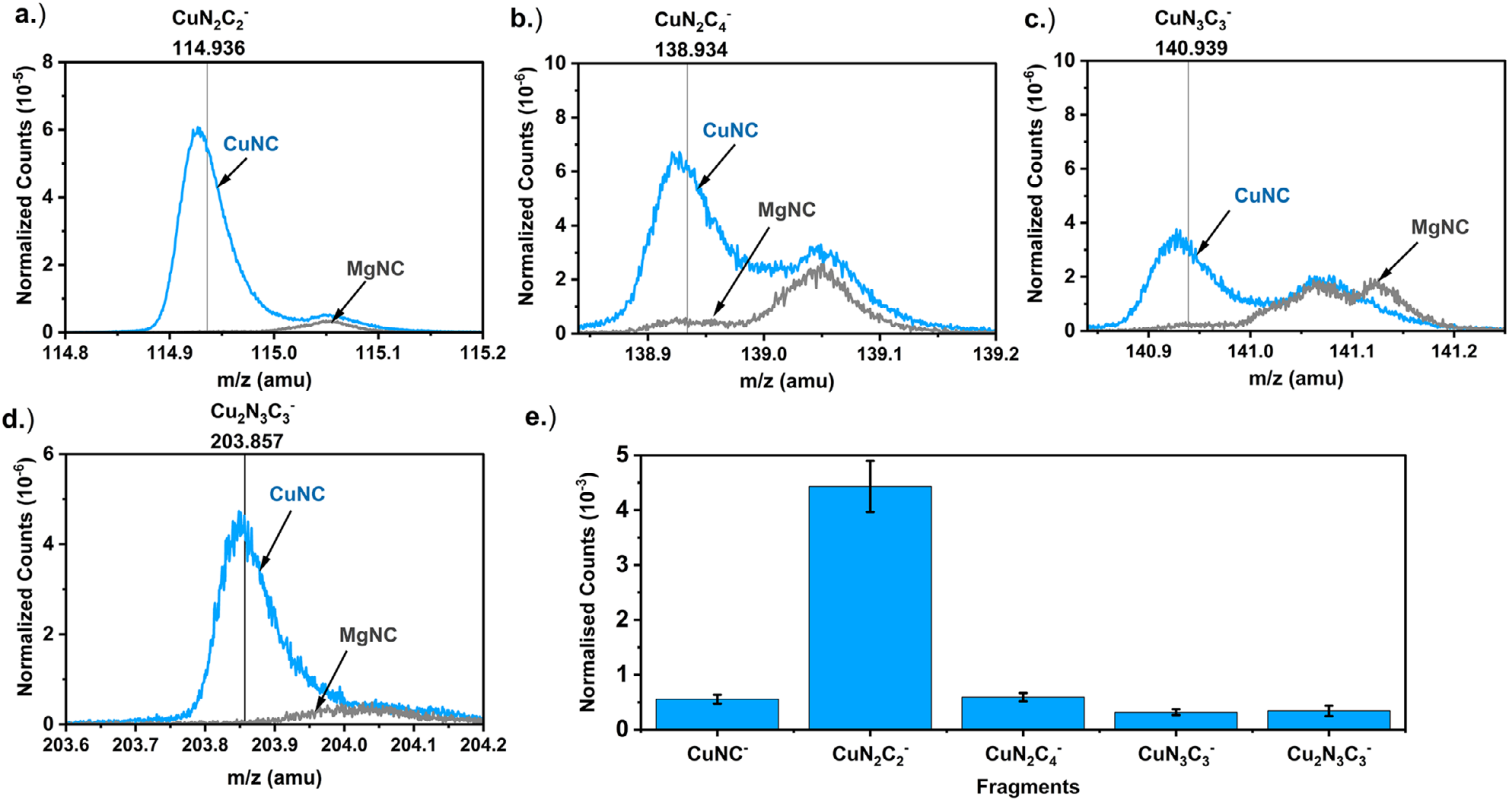
ToF‐SIMS Cu‐based fragments in CuNC and MgNC in negative polarity SIMS spectrum shown for CuN_2_C_2_^−^ (a) CuN_2_C_4_^−^ (b) CuN_3_C_3_^−^ (c) Cu_2_N_3_C_3_^−^ (d) and list of identified major Cu‐based fragments (e). Error bars represent standard deviation from four repeats in separate locations. A Bi^3+^ primary ion beam over 100 µm × 100 µm field of view with 25 keV and 0.4 pA beam current was used, while the sputter beam consisted of a 11 nA Ar beam (≈1600 cluster size) at 10 keV rastered over 500 µm × 500 µm with non‐interlaced 1 sputter frames and 1 s pause, up to a dose density 10^15^ ions·cm^−2^. Fragment counts from peak areas were normalized to the total ion counts.

For further characterizations on the atomic dispersion of FeNC, NiNC, CoNC, and CrNC, we would like to refer the reader to our previous reports, from which we prepared the materials following the same synthetic approach. These works demonstrate the atomic metal dispersion via ^57^Fe Mossbauer Spectroscopy, X‐ray Absorption Spectroscopy (XAS), Time‐of‐Flight Secondary Ion Mass Spectrometry (ToF‐SIMS), High‐Angle Annular Dark Field Scanning Transmission Electron Microscopy (HAADF‐STEM) and Energy‐Dispersive X‐ray Spectroscopy (EDXS).^[^ ^54^, ^55^, ^57^ ^]^ We previously observed that the low‐temperature Mg‐exchange protocol leads to mainly tetracoordinated Fe^3+^ and Ni^2+^ atomically dispersed single sites with pyridinic coordination; in the case of Fe^3+^, an axial ligand was also observed, which is expected to be stripped and replaced by the relevant chemical species involved in the electrocatalytic measurements. The atomic dispersion of the active metal in CrNC have been previously assessed by means of ToF‐SIMS, HAADF‐STEM and EDXS, while CoNC was evaluated with ToF‐SIMS.^[^ ^57^ ^]^ Here, CuNC was synthesized for the first time and its atomic dispersion is evidenced by ToF‐SIMS in negative polarity (**Figure** **2**). For comparison, ToF‐SIMS of MgNC was also measured, with the normalized mass spectrums of CuNC and MgNC from 50‐250 amu shown in Figure S6 (Supporting Information). Various peaks present in the mass spectrum of CuNC and absent in MgNC were identified, with these peaks appearing at m/z equivalent to ion fragments consisting of single atoms of Cu with N_x_C_y_ (x and y = 1‐4, Figure 2a–c). The most prevalent Cu‐based ion fragment was CuN_2_C_2_^−^ (Figure 2e). Previous ToF‐SIMS measurements on our synthesized MNC (M = Co, Cr, Ni) also found MN_2_C_2_ as the most significant M‐containing peak.^[^ ^54^, ^55^, ^57^ ^]^ It should be made clear that the ion fragment counts are dependent on the ionization efficiency (secondary ion formation and survival probability),^[^ ^61^ ^]^ which will vary for each fragment. Additionally, ToF‐SIMS only provides purely chemical information, meaning the results do not provide coordination information (e.g. N_2_‐Cu‐C_2_). Based on our previous reports and those of others, it is most likely that single Cu atoms are coordinated to N_x_ (x = 1‐5), within a C framework. Interestingly, an ion fragment containing two Cu atoms, assigned here as Cu_2_N_3_C_3_^−^, was also observed (Figure 2d). As prepared and pyrolyzed Co phthalocyanine has also previously shown Co_2_N_3_C_3_ fragments,^[^ ^62^, ^63^ ^]^ with Dodelet and coworkers tentatively assigning this to a µ‐cyano‐dimer.^60^ No peaks in the mass spectrum were found corresponding to fragments containing ≥ three Cu atoms, indicating a purely atomic distribution of Cu. Chemical maps of the assigned fragments also evidenced a homogenous dispersion of Cu throughout the CuNC (Figure S7, Supporting Information).

### 2.2 Electrochemical Performance

Nitrate electroreduction was conducted in a custom‐built H‐shaped electrochemical cell (Figure S8, Supporting Information) at room temperature under a controlled Argon (White Martins, 99.999%) flow/atmosphere. The experiments used 0.1 mol·L^−1^ NaOH (pH 13, Sigma‐Aldrich, 99.6%) with 0.5 mol·L^−1^ NaNO_3_ (Sigma‐Aldrich, ≥ 99,0%) as the electrolyte, and the potential was fine‐tuned in situ with 85% of the uncompensated resistance (R_u_) correction to minimize potential losses. A graphite bar served as the auxiliary electrode, a reversible hydrogen electrode made in 0.1 mol·L^−1^ NaOH was used as the reference electrode, and the working electrode, with a geometric area of 0.25 cm^2^, consisted of catalyst ink deposited on carbon cloth (CeTech Carbon Cloth W1S1010) at a fixed ratio of 0.5 mg_MNC_·cm^−2^ (see experimental section in SI for further details).

**Figure 3:**
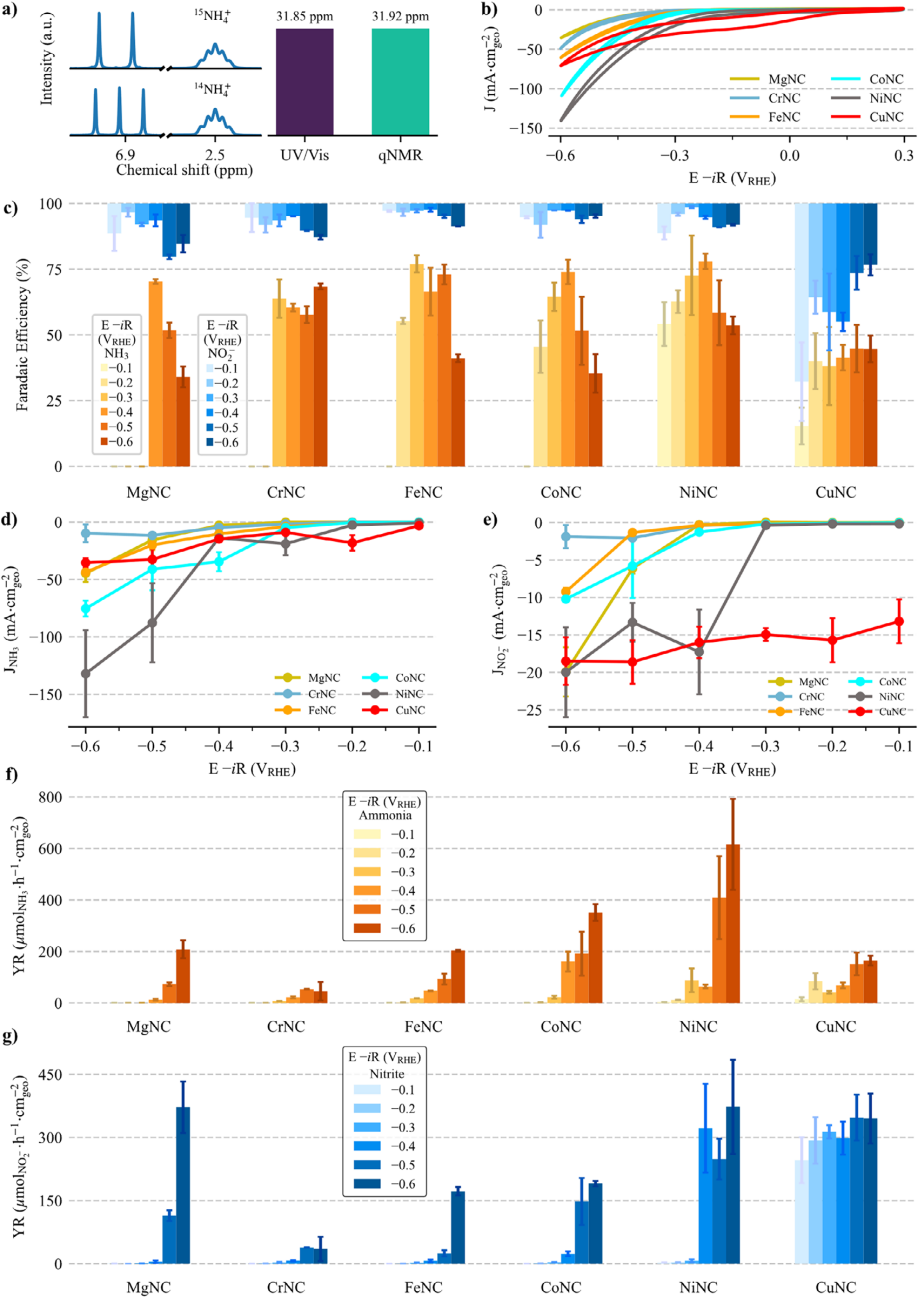
^1^H NMR with ^15^NH_4_^+^ (two peaks) and ^14^NH_4_^+^ (three peaks), with DMSO as reference in 2.5 ppm, and a comparison of quantification with ^1^H NMR and UV/Vis techniques (a), cyclic voltammetry at 20 mV·s^−1^ from +0.3 to −0.6 V_RHE_ (b), FE for ammonia and nitrite (c), partial current density for ammonia and nitrite (d and e, respectively), and yield rate for ammonia and nitrite (f and g, respectively), where each electrochemical experiment was measured at least twice with 85% iR compensation in 0.1 mol·L^−1^ NaOH + 0.5 mol·L^−1^ NaNO_3_ with Ar 99.999% as purging gas in a H‐type cell with a graphite bar as CE and a Fumasep membrane to separate the compartments and the error bars are the standard deviation of the measurements. Electrolysis was conducted for 1‐h.

Initially, electrolysis was performed at −0.6 V_RHE_ with both labeled nitrate isotope (^15^NO_3_Na, Sigma‐Aldrich, 98% ^15^N) and the most abundant isotope (^14^NO_3_^−^) to verify the system's isolation from external nitrogen sources and to ensure the reliability of the UV/Vis spectrophotometry method for detecting and quantifying ammonia method (see Figures S9–S12, Supporting Information and experimental section in Supporting Information for further details). ^1^H NMR spectroscopy was utilized, confirming system isolation and the quantification accuracy between UV/Vis and ^1^H NMR methods with 99.8% precision (**Figure** **3**a). Figure 3a depicts two spectra: one attributed to ^15^NH_4_^+^ species, showing a doublet, and another to ^14^NH_4_^+^, displaying a characteristic triplet,^[^ ^64^, ^65^ ^]^ the quintuplet at 2.5 ppm is from DMSO‐*d_6_* (Sigma‐Aldrich, 99.9% D) that was used as reference. This difference between the isotopes arises from the distinct magnetic spin values of ^14^N and ^15^N nuclei, which are 1 and 0.5, respectively. These results confirm that the NH_3_ generated by eNO_3_RR originates from the nitrate source in the solution, rather than other source such as NO_x_ contaminants or N from the matrix of the MNC.^[^ ^44^, ^66^, ^67^ ^]^

Figure 3b presents the cyclic voltammetry profiles for all the metals studied (see Figures S13–S15, Supporting Information for additional comparison). Among these, the CuNC profile stands out with its onset potential (E_onset_); in this work defined as the potential at which a current density of −0.4 mA·cm^−2^ was achieved,^[^ ^14^ ^]^ at +0.25 V_RHE_, earlier than the other metals. The peak near −0.1 V_RHE_ may be due to the reduction of nitrate to nitrite, as suggested by Liu et al.,^[^ ^68^, ^69^ ^]^ followed by the beginning of a hydrogen evolution profile. The other metals show E_onset_ at more negative potentials: NiNC at +0.2 V_RHE_, CoNC at −0.25 V_RHE_, FeNC at −0.18 V_RHE_, CrNC at −0.1 V_RHE_, and MgNC at −0.1 V_RHE_. Despite its early onset potential, Cu does not exhibit the exponential current increase seen in other metals. This is likely due to Cu's higher affinity for forming nitrite intermediates rather than ammonia, as well as the selectivity toward H_2_ production (Figure 3c). This observation suggests a different reaction mechanism for CuNC compared to the other metals, highlighting its potential for selective and efficient nitrite production.

In Figure 3c all transition metals except CuNC achieved faradaic efficiencies for ammonia production ($FE_{NH_{3}}$) exceeding 50%. MgNC was used in this work as a blank control allowing the performance of the active transition metal sites to be distinguished from that of the N‐doped carbon support, also probably due to nitrogen moieties^[^ ^70^, ^71^ ^]^ (only trace Mg was left (Figures S1 and S3, Supporting Information) and Mg lacks occupied *d* orbitals to effectively participate in the redox steps of the nitrate reduction pathway), surpassing 50% $FE_{NH_{3}}$ at −0.4 V_RHE_. Although it decreased at more negative potentials, likely due to competing hydrogen evolution reaction (HER); FE for nitrite production ($FE_{{\mathrm{NO}}_{2}^{-}}$) never exceeded 25% at any potential. CrNC, in contrast, demonstrated consistent $FE_{NH_{3}}$ starting at −0.3 V_RHE_, maintaining above 50% at all potentials, with a maximum of 68.4 ± 1.1% at −0.6 V_RHE_ and a low $FE_{{\mathrm{NO}}_{2}^{-}}$, similar to the other metals (except CuNC). FeNC displayed $FE_{NH_{3}}$ beginning at −0.2 V_RHE_, reaching 77.0 ± 3.3% at −0.3 V_RHE_, consistent with the previous report by Wu et al.^[^ ^44^ ^]^ This high selectivity was maintained until −0.6 V_RHE_, at which point $FE_{NH_{3}}$ dropped below 50%. CoNC followed a similar trend, with $FE_{NH_{3}}$ starting at −0.2 V_RHE_ and peaking 74.0 ± 4.6% at −0.4 V_RHE_ before declining at more negative potentials. Notably, NiNC outperformed the other metals, achieving over 50% $FE_{NH_{3}}$ as early as −0.1 V_RHE_, with a peak efficiency of 78.0 ± 2.9% at −0.4 V_RHE_. Despite competing with hydrogen evolution, NiNC remained above 50% even at more negative potentials. In contrast, CuNC, while efficient for nitrite production, never exceeded 50% efficiency for ammonia production, highlighting its selectivity for nitrite formation: $FE_{{\mathrm{NO}}_{2}^{-}}$ = 67.8 ± 14.9% at −0.1 V_RHE_.

In Figure 3c a clear trend in overpotential (η)—in this work defined by the difference of the thermodynamic potential and the first applied potential to have a significant amount of ammonia produced—required for ammonia production is evident, reflecting the intrinsic activity differences among the catalysts. MgNC exhibits limited activity and only begins producing ammonia at η = 1.11 V (−0.4 V_RHE_), likely influenced by nitrogen moieties in the support. CrNC follows with η = 1.01 V (−0.3 V_RHE_), while FeNC and CoNC perform with onset values around η = 0.91 V (−0.2 V_RHE_). NiNC and CuNC demonstrate the lowest overpotentials, initiating ammonia production at η = 0.81 V (−0.1 V_RHE_), indicating a more favorable energetic pathway. Compared to other reports in the literature, the metal centers examined in this work that overlap with those studied by Murphy et al.^[^ ^14^ ^]^ exhibited similar performance: all metals except Co had an overpotential of 0.98 V, while cobalt showed a higher overpotential of 1.18 V. Li and Wu's papers,^[^ ^44^, ^45^ ^]^ which focused specifically on FeNC catalysts, achieved overpotentials of 0.91 V and 1.28 V, respectively. NiNC and CuNC, presented in this work, exhibit the lowest overpotentials. It is noted that since the equilibrium potential for eNO_3_RR changes with pH, comparisons of overpotentials between different studies should be made carefully.

Some studies in the literature have investigated eNO_3_RR at different pH values, from near‐neutral^[^ ^14^, ^44^ ^]^ to highly alkaline conditions.^[^ ^45^ ^]^ Direct comparison between these studies is not straightforward, as the thermodynamic potential for eNO_3_RR is inherently pH‐dependent. Unlike reactions with a 1:1 proton‐to‐electron transfer ratio, eNO_3_RR involves higher proton‐to‐electron ratios (e.g., 10/8 or 9/8), causing the equilibrium potential to shift with pH even when referenced to the RHE scale. As a result, the equilibrium potential decreases from 0.79 V_RHE_ at pH 6 to 0.71 V_RHE_ at pH 13, meaning that applying the same potential *vs*. RHE at different pH values corresponds to different overpotentials. Moreover, under alkaline conditions, an electrocatalyst with high selectivity to NH_3_ will experience a greater pH polarization compared to a NO_2_^−^ and H_2_ selective catalysts due to the different number of OH produced per electron.^[^ ^72^ ^]^ Furthermore, the dominant product changes with pH: below the pKa of ammonium (9.25 at 25 °C), NH_4_^+^ is the favored product, while above the pKa, NH_3_ becomes dominant. This leads to different half‐reactions with distinct proton requirements and Nernstian slopes,^[^ ^22^ ^]^ further complicating comparisons across pH. Additionally, high current density and low electrolyte flow conditions can cause distinct and local pH gradients around the catalyst interface, especially in non‐buffered electrolytes.^[^ ^73^ ^]^ Different pH can lead to different selectivity from HER competition (lower HER at high pH)^[^ ^74^ ^]^ or intermediates adsorption and poisoning (facile binding of NO_2_^−^* and H^+^ at low pH).^[^ ^73^ ^]^ Therefore, pH effects must be carefully considered when benchmarking eNO_3_RR catalysts. For further discussion on the effect of pH and ionic strength on eNO3RR, we refer the reader to the review by Fan et al.^[^ ^75^ ^]^

The partial current density (Figure 3d,e, see Supporting Information for further details) corroborates these trends. At −0.5 V_RHE_, NiNC exhibits an ammonia partial current density ($J_{NH_{3}}$) close to −90 mA·${\mathrm{cm}}_{\mathrm{geo}}^{-2}$ (Figure 3d), demonstrating its high catalytic activity. However, its $FE_{NH_{3}}$ dropped to ≈50% due to the start of parallel reactions, which can be due to HER and N‐containing by‐products. In contrast, the $FE_{{\mathrm{NO}}_{2}^{-}}$ remained below 10%, and the nitrite partial current density ($J_{{\mathrm{NO}}_{2}^{-}}$) did not exceed −20 mA·${\mathrm{cm}}_{\mathrm{geo}}^{-2}$ (Figure 3e). Even at more negative potentials, NiNC maintained higher current densities, reaching ≈−130 mA·${\mathrm{cm}}_{\mathrm{geo}}^{-2}$ at −0.6 V_RHE_. In contrast, the other metals do not exceed −50 mA·${\mathrm{cm}}_{\mathrm{geo}}^{-2}$, even at more negative potentials, except for CoNC, which achieved −75.3 ± 6.9 mA·${\mathrm{cm}}_{\mathrm{geo}}^{-2}$ at −0.6 V_RHE_. Regarding $J_{{\mathrm{NO}}_{2}^{-}}$, the metals showed profiles similar to those for $J_{NH_{3}}$, with two exceptions. MgNC demonstrates an increase in $J_{{\mathrm{NO}}_{2}^{-}}$, and CuNC, which maintains a constant $J_{{\mathrm{NO}}_{2}^{-}}$ between −15 and −20 mA·${\mathrm{cm}}_{\mathrm{geo}}^{-2}$, nearly double that of the other metals.

Figure 3f,g shows the yield rates for ammonia and nitrite, respectively. A distinct trend emerges across the metals, with increasing ammonia production from CrNC to NiNC, though CuNC consistently deviates from this pattern. Ammonia production starts below 100 µmol·h^−1^·${\mathrm{cm}}_{\mathrm{geo}}^{-2}$ with CrNC at its optimum potential (–0.5 V_RHE_), rises 200 µmol·h^−1^·${\mathrm{cm}}_{\mathrm{geo}}^{-2}$ for FeNC in –0.6 V_RHE_, and reaches 351.4 ± 32.4 µmol·h^−1^·${\mathrm{cm}}_{\mathrm{geo}}^{-2}$ for CoNC at –0.6 V_RHE_. FeNC and MgNC exhibit comparable yield rates to ammonia ($YR_{NH_{3}}$) in the –0.4 to –0.6 V_RHE_ range, contrasted by a significant difference in nitrite yield rate ($YR_{{\mathrm{NO}}_{2}^{-}}$), where FeNC maintains a consistently low $YR_{{\mathrm{NO}}_{2}^{-}}$ across nearly all potentials and only shows a notable increase at –0.6 V_RHE_. Meanwhile, MgNC exhibits a much higher $YR_{{\mathrm{NO}}_{2}^{-}}$, exceeding 100 µmol·h^−1^·${\mathrm{cm}}_{\mathrm{geo}}^{-2}$ at –0.5 V_RHE_, indicating that FeNC is more selective toward ammonia under these conditions. NiNC shows the best yield among the studied materials at potentials below −0.5 V_RHE_, achieving 615.7 ± 176.5 µmol·h^−1^·${\mathrm{cm}}_{\mathrm{geo}}^{-2}$ at −0.6 V_RHE_. Xi et al.^[^ ^42^ ^]^ reported work with NiNC SACs combined with Ni nanoparticles on carbon nanotubes grown on carbon cloth, which showed similar results to ours despite the discrepancy in substrates and pH. Xi et al. reported efficiencies of ≈75% and yields in the mmol range—potentially indicative of a similar mechanistic scenario, albeit under significantly more cathodic applied potentials.^[^ ^42^ ^]^ In contrast, CuNC, despite its early significant current, produced nearly as little ammonia as CrNC, but reached ≈200 µmol·h^−1^·${\mathrm{cm}}_{\mathrm{geo}}^{-2}$ at more negative potentials. CuNC produced nearly 300 µmol·h^−1^·${\mathrm{cm}}_{\mathrm{geo}}^{-2}$ of nitrite at all potentials, while the other metals only show significant nitrite production at the most negative potentials. In bulk Cu electrodes, considerable amounts of nitrite are commonly detected as an intermediate.^[^ ^76^, ^77^, ^78^ ^]^ This suggests that CuNC likely produced Cu nanoparticles under these reducing conditions, as previously observed during nitrite reduction.^[^ ^47^ ^]^ This is in contrast to Zhu et al. work on Cu SACs, which did not show a significant presence of NO_2_^−^.^[^ ^46^ ^]^ On the other hand, Yang et al. showed that it was the nanoparticles formed from the copper single‐atom that produced ammonia in their system due to the application of the potential, and this was corroborated by DFT, which indicated that their single‐atoms would not be able to overcome the energy barrier of converting nitrate into ammonia, stagnating the reduction in nitrite.^[^ ^47^, ^67^ ^]^

**Figure 4:**
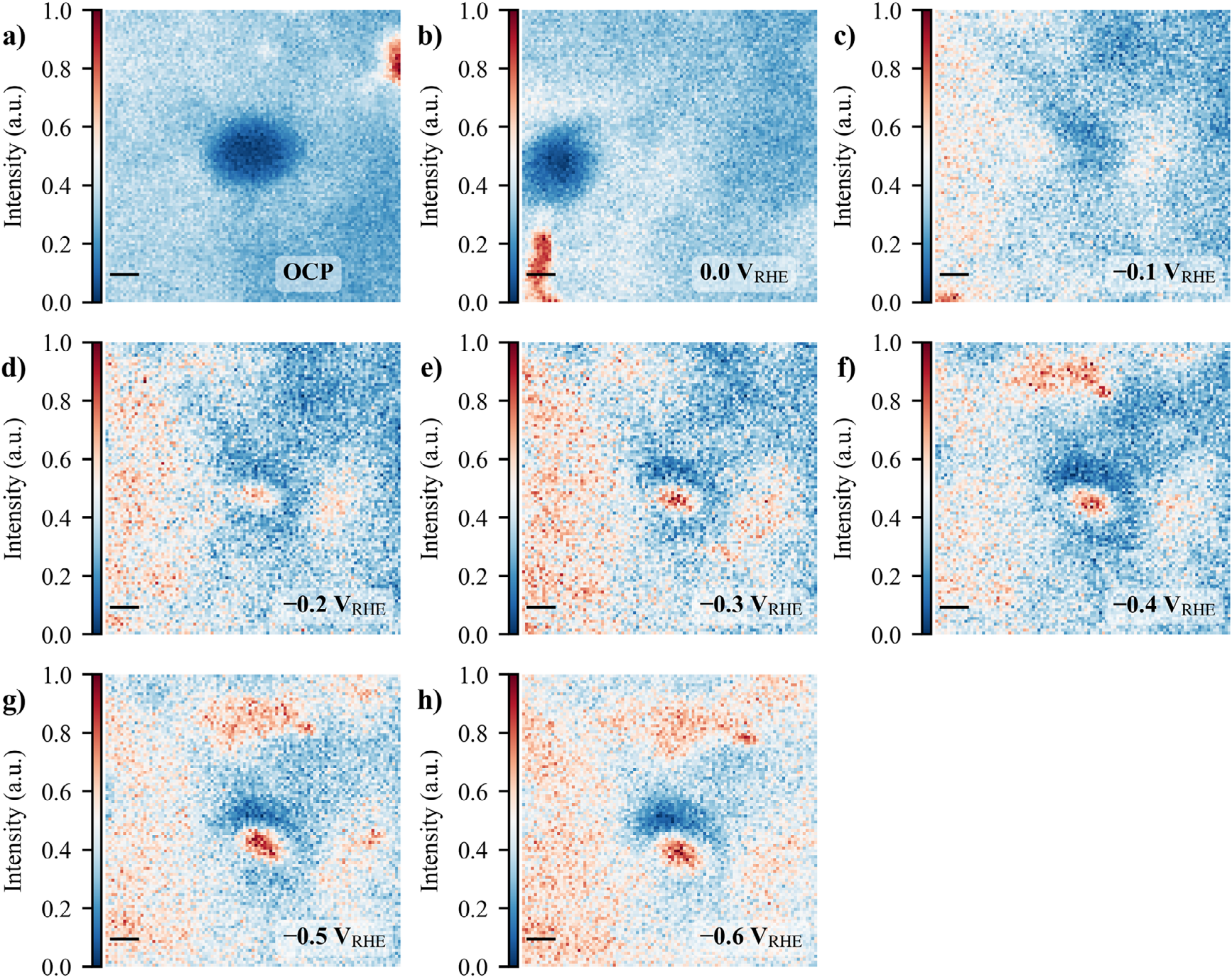
In situ SXRF for CoNC in a trajectory 10 µm x 10 µm at OCP of 0.15 V_RHE_ (a), and then from 0.0 to −0.6 V_RHE_ (b‐h, with a step of −0.1 V). The scale bar in each map is of 1 µm. The maps were obtained using an excitation energy of 6931 eV and the Co Kα emission lines, therefore representing the material distribution over the substrate. The in situ SXRF electrochemical experiment was measured without *i*R compensation in 0.1 mol·L^−1^ NaOH + 0.5 mol·L^−1^ NaNO_3_ and the catalyst loading was 0.5 mg·cm^−1^.

**Figure 5:**
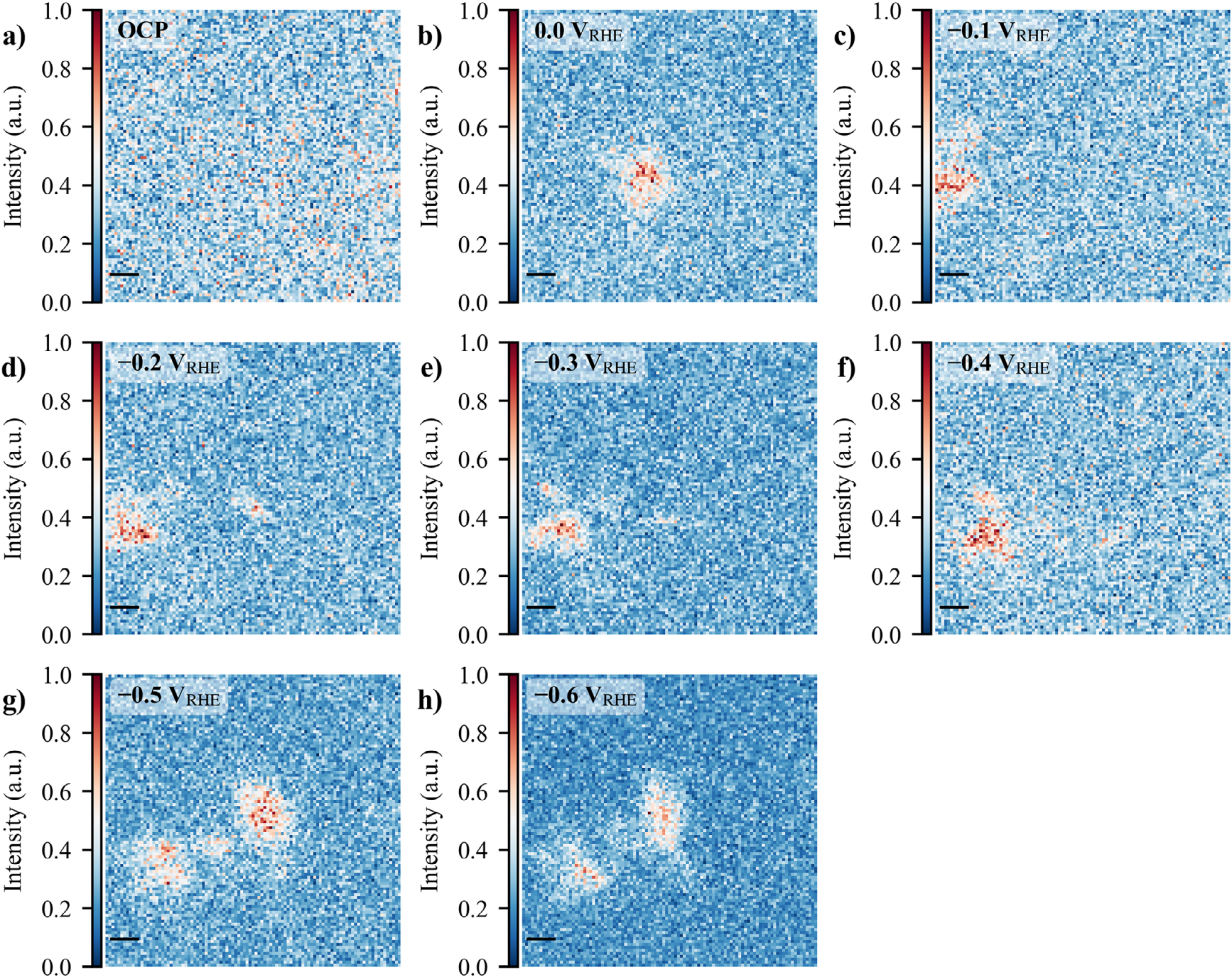
In situ SXRF for NiNC in a trajectory of 10 µm × 10 µm at OCP of 0.15 V_RHE_ (a), and then from 0.0 to −0.6 V_RHE_ (b‐h, with a step of −0.1 V). The scale bar in each map is of 1 µm. The maps were obtained using an excitation energy of 7480 eV and the Ni Kα emission lines, thus representing the material distribution over the substrate. The in situ SXRF electrochemical experiment was measured without *i*R compensation in 0.1 mol·L^−1^ NaOH + 0.5 mol·L^−1^ NaNO_3_, and the catalyst loading was 0.5 mg·cm^−2^.

Ex situ characterization on post‐electrolysis SAC systems is challenging due to the dynamic evolution of the structure. Yang et al.^[^ ^47^ ^]^ and our previous work^[^ ^58^ ^]^ showed that such catalysts can evolve from single atoms to nanoclusters under operating conditions and this potential‐driven transformation is likely reversible or metastable. Therefore, switching off the electrochemical potential for *post‐mortem* characterization could cause the active clusters to re‐disperse, leading to unrepresentative conditions of the catalytic state and possible incorrect conclusions about structure‐activity‐stability relationships. Then, in addition to the electrochemical experiments, in situ SXRF measurements (see Figure S17, Supporting Information and Supporting Information for further details) were conducted on both CoNC (**Figure** **4**) and NiNC (**Figure** **5**) to further elucidate the spatial distribution and behavior of metal sites during eNO_3_RR. These high‐resolution elemental maps provide insights into how the metal is distributed across the carbon‐nitrogen matrix at different potentials and how their arrangement evolves under electrochemical conditions. Figure 4a–h, and Figure 5a–h, illustrate the formation of cobalt and nickel clusters, respectively, as the potential becomes more reducing. At OCP (0.15 V_RHE_), CoNC exhibits the presence of Co particles, a feature also observed at 0.0 V_RHE_. This clustering may be attributed to storage effects and the electrode ink fabrication process.^[^ ^79^ ^]^ Notably, there is a widespread distribution of cobalt‐rich regions throughout the N–C matrix, as evidenced by the intense white coloration in the elemental maps. As the applied potential becomes more negative, these white regions transition to red, as the Co signal intensity increases due to the potential atomic migration. This dynamic behavior is further illustrated by the shifting position of a central void (represented by a blue region) from the center at OCP to the left at 0.0 V_RHE_. At –0.2 V_RHE_, initial signs of Co agglomeration appear in the central region of the SXRF map. These agglomerates become more pronounced at increasingly negative potentials. This morphological evolution correlates with the observed rise in ammonia FE from –0.2 to –0.4 V_RHE_. However, a sudden decrease in FE at more negative potentials may indicate the rise of competing side reactions and the formation of larger Co clusters, potentially reducing the selectivity toward ammonia production.

At OCP (0.15 V_RHE_), Figure 5a, Ni is evenly dispersed across the carbon‐nitrogen matrix. However, starting from 0.0 V_RHE_ (Figure 5b) to −0.4 V_RHE_ (Figure 5f), Ni begins to aggregate, forming isolated clusters. At −0.5 and −0.6 V_RHE_ (Figure 5g,h), the SXRF maps reveal the emergence of two well‐defined clusters. This may be influenced by parallel reactions, including the HER, which produces gas bubbles that may displace metal atoms on the catalyst surface.

The SXRF maps align with the electrochemical trends observed for NiNC. As previously noted, NiNC exhibited over 50% $FE_{NH_{3}}$ as early as −0.1 V_RHE_, with a peak of 78.0 ± 2.9% at −0.4 V_RHE_. The fragmentation behavior observed in Figure 5g,h matches with the decrease in $FE_{NH_{3}}$ at −0.5 to −0.6 V_RHE_, likely due to HER and other parallel reactions, which alters the distribution and availability of active Ni sites for nitrate reduction. Similarly, the current density profiles at these potentials show a significant increase in $J_{NH_{3}}$, exceeding −150 mA·${\mathrm{cm}}_{\mathrm{geo}}^{-2}$ at −0.4 V_RHE_, before plateauing and slightly decreasing at more negative potentials. This behavior is mirrored in the SXRF data, where the Ni clusters appear to undergo leaching, potentially limiting the catalyst's ability to sustain high $FE_{NH_{3}}$. At pH 13, as used here, 0.0 V_RHE_ equates to −0.77 V_SHE_, which on the experimentally derived Ni Pourbaix diagram is the region where the transition from Ni(OH)_2_/Ni(OH)_3_^−^ to Ni occurs.^[^ ^80^ ^]^ As a more negative potential is applied, Ni becomes the thermodynamically favored species from its Pourbaix diagram. However, with a more reductive potential, the current density to NH_3_ and H_2_ increases, causing a small increase in local pH from OH^−^ produced and H^+^ consumed, respectively. Dissolution of Ni from NiNC may occur under both OCP and reductive conditions, as previously found from *online* ICP‐MS studies of our FeNC for O_2_ reduction.^[^ ^58^ ^]^ Metal dissolution and change in active sites of MNCs could also occur during ink preparation and storage and electrode formation.^[^ ^79^ ^]^ Dissolved Ni species could then result in the active Ni cluster formation observed. This potential‐dependent clustering observed, and its subsequent impact on eNO_3_RR performance, contrasts with the observations made for ZIF‐8 derived NiNC during CO_2_RR by Cuenya and coworkers, where such instability was not observed.^[^ ^49^ ^]^ In the CO_2_RR experiments (CO_2_‐saturated 0.1 mol·L^−1^ KHCO_3_ solution), no such Ni cluster formation was observed even at highly negative potentials (−1.15 V_RHE_, ≈−28 mA·cm^−2^), based on *operando* XAS. As discussed in the introduction, such ZIF‐8 derived MNC typically exhibit electrochemical active site utilization < 10%, based on five electron in situ nitrite reduction.^[^ ^50^, ^54^ ^]^ Furthermore, the ZIF‐8 derived NiNC catalyst maintained a high FE for CO (> 90%) throughout the investigated potential window, showing no decline at more negative potentials, unlike our case under eNO_3_RR. Here, $FE_{NH_{3}}$ for our NiNC falls from 77 and 72% at −0.4 and −0.3 V_RHE_ to 17 and 24%, respectively, after 10‐h (Figure S118, Supporting Information). This difference underscores the possible significant impact of the reactant (NO_3_^−^ *vs*. CO_2_), storage conditions, synthesis pathway and characterization technique conditions on the observed MNC SAC structural evolution and consequently its performance and stability.

In the case of CoNC, reversible metallic cluster formation was observed by Cuenya and coworkers during CO_2_RR using *operando* XAS,^[^ ^49^ ^]^ with similar cluster formation also seen during eNO_3_RR, as shown in Figure 4, suggesting a greater mobility of Co compared to Ni in both reaction environments, although the cluster formation kinetics might be different.

### 2.3 Theoretical Calculations

The adsorption energy of reactants serves as a crucial descriptor for understanding and predicting catalytic activity for electrochemical reactions. We use two different models of MNCs containing pyridinic and pyrrolic N‐atoms to calculate the adsorption energies. To validate the model, first we evaluated the H*‐binding energy on the surfaces and compared it to the HER volcano plot with respect to the HER onset potential (potential at 5 mA·cm^−2^).^[^ ^81^ ^]^ The results, shown in Figure S18 (Supporting Information), indicate that early transition metals like Cr, Fe, and Co align well with the volcano plot, compared to Cu and Ni. Furthermore, the metallic form of Ni (FCC (111)) demonstrates a strong fit in the volcano plot, suggesting that the HER activity predominantly arises from the metal cluster.

**Figure 6:**
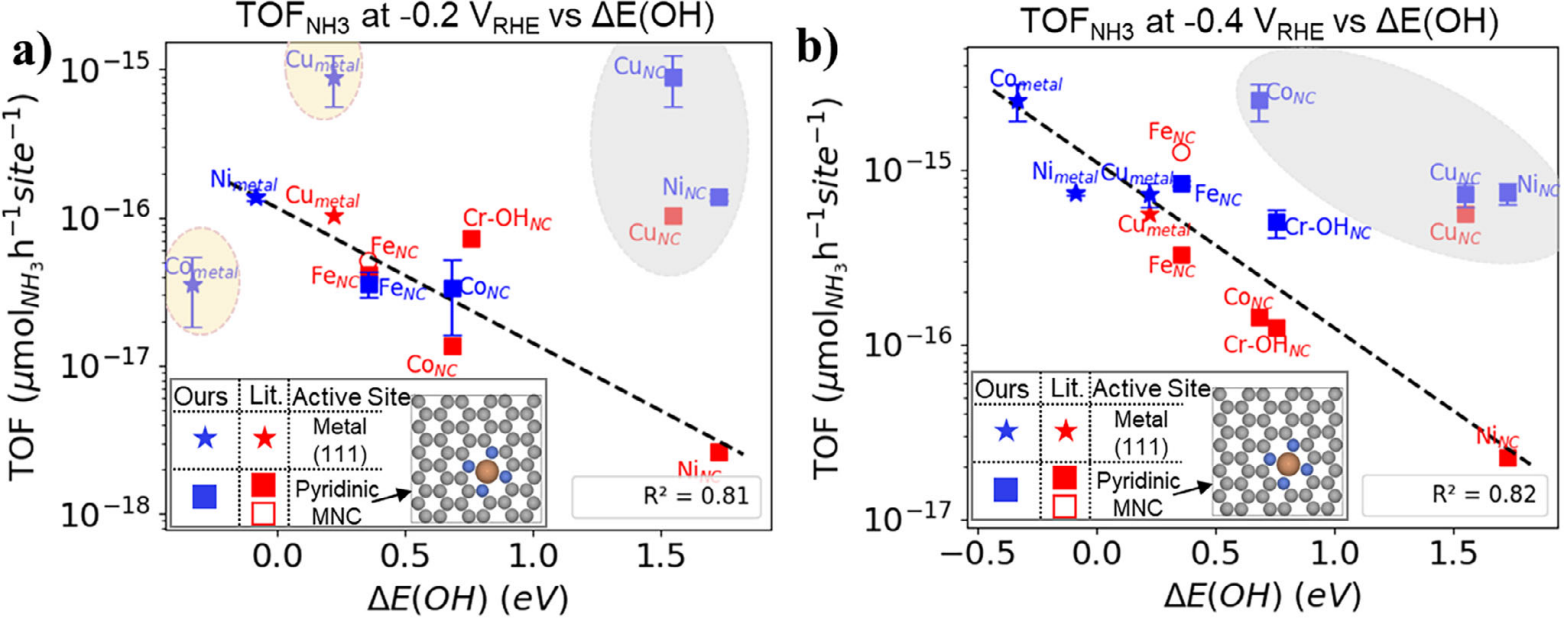
Correlation between *OH adsorption energy and TOF (µmol_NH3_·h^−1^·site^−1^) for pyridinic MNC and metal surfaces (FCC (1 1 1)) at a.) −0.2 V_RHE_ (a). −0.4 V_RHE_ (b). Our data is shown in blue (0.1 mol·L^−1^ NaOH + 0.5 mol·L^−1^ NaNO_3_, 1‐h at applied potential) and TOF calculated from Murphy et al. (0.05 mol·L^−1^ mol·L^−1^ PBS + 0.16 mol·L^−1^ KNO_3_ electrolyte, 2‐h at applied potential) in filled red,^[^ ^14^ ^]^ assuming all metal from ICP‐MS is coordinated as MNC with 100% electrochemical active site utilization. The hollow symbols for FeNC TOF data is taken from Li et al.^[^ ^45^ ^]^ (0.1 mol·L^−1^KOH and 0.1 mol·L^−1^KNO_3_ electrolyte, 30 min at applied potential), where TOF was obtained by Surface Interrogation Scanning Electrochemical Microscopy. The inset shows the pyridinic computational models used for MNCs. Stars represent metal surfaces for Ni, Co and Cu. The data points in yellow and grey regions were not used in the calculation of R^2^. Error bars correspond to at least two measurements.

Next, we use the adsorption trend of the reactant over the MNCs to understand their activity. NO_3_^−^ binds to the surface via the O‐atom and shows a linear relation with the adsorption energy of other chemical species that bind through the O‐atom, like *OH.^[^ ^82^ ^]^ Therefore, for simplicity, we use the *OH adsorption energy as a descriptor to understand the catalytic activity of NO_3_^−^ reduction to NH_3_. The correlation between the *OH adsorption energy and the TOF to NH_3_ at −0.2 and −0.4 V_RHE_ is shown in **Figure** **6** for pyridinic‐N and Figure S19 (Supporting Information) for pyrrolic and pyridinic‐N. To substantiate our result, we include previous experimental results on MNC catalysts from Murphy et al.^[^ ^14^ ^]^ and Li et al.^[^ ^45^ ^]^ The TOF for our data and for data from Murphy et al. was calculated based on the active site density from ICP‐MS, assuming all metal participants as active sites (see Supplementary Information), therefore providing a minimum TOF. The TOF of FeNC from Li et al. is obtained directly from their site density measurements using Surface Interrogation Scanning Electrochemical Microscopy (SI‐SECM).^[^ ^45^ ^]^ It is noted that the true electrochemical active site density, and therefore TOF, likely deviates from those reported here based on ICP‐MS. Although, in the case of FeNC there is little deviation between the TOF derived from ICP‐MS and the TOF measured directly by Li et al.^[^ ^45^ ^]^ from SI‐SECM (≈2‐4·10^−17^ $\mu \mathrm{mo}l_{NH_{3}}$·h^−1^·site^−1^ at −0.2 V_RHE_). In any case, the objective here is to capture the structure‐activity trend, which is feasible based active site density from ICP‐MS.

As can be seen in Figure 6a, Figure S19a,c (Supporting Information), there is a clear trend between the *OH adsorption energy and the TOF at −0.2 V_RHE_ on pyrrolic and pyridinic MNCs except for CuNC and our NiNC. Instead, Ni_metal_ fits well with our trend, which matches our SXRF observations of Ni clustering (Figure 5). The possible reasons for our NiNC instability were discussed earlier. Additionally, as previously discussed, CuNC are known to form Cu clusters and nanoparticles under reducing conditions. The higher TOF for our Cu_metal_ compared to Murphy et al.^[^ ^14^ ^]^ may arise from the different accessibility of Cu in our highly porous catalyst, or an alternative Cu phase being present. Aside from this, our TOFs are quite comparable to those reported in literature, suggesting that the catalytic activity is primarily associated with most MNC metal centers. For CrNC, the adsorption energy aligns more effectively when the metal center is back‐bonded to an OH‐ligand (Cr‐OH), which can be attributed to the highly oxyphilic and reactive nature of the Cr center.^[^ ^57^ ^]^ To understand the stability of Cr‐NC with back ligand we calculated the adsorption energy of OH and O on Cr‐NC and compared with Fe‐NC (Figure S20, Supporting Information). The calculations show that both *OH and *O are stable by −0.39 and −0.11 eV respectively for CrNC (pyridinic‐N) with reference to free water. The energetics suggest that CrNC with OH‐ligand could be stable at lower reducing potentials. An additional way to consider the stability of the metal center is through its formation energy. The formation energy for the MNCs, calculated with reference to the corresponding metal counterpart (Figure S21, Supporting Information), decreases from Cr to Cu. This decreasing trend suggests that metals in MNCs become increasingly prone to clustering as one moves from Cr to Cu, due to the lower formation energy.

Interestingly, at −0.4 V_RHE_ (Figure 6b; Figure S19b,d, Supporting Information) our CoNC TOF does not fit the observed trend and displays a substantially weaker binding energy toward *OH compared to Co_metal_, which fits well with the TOF. This observation suggests that Co's activity likely originates from Co_metal_ at more reducing potentials than −0.2 V_RHE_. In this regard, Stoerzinger and coworkers found Co foil was highly selective to ammonium.^[^ ^83^ ^]^ The presence of Co particles under more reducing conditions mirrors the in situ SXRF results, where clustering intensifies below −0.2 V_RHE_ (Figure 4). From the stability measurements at −0.4 V_RHE_, an increase in current density from −20 to −30 mA·cm^−2^, with maintained FE to ammonia (80‐72%), was observed for CoNC from 1–2‐h, respectively (Figure S17, Supporting Information). This suggests that the more active Co_metal_ phase was being formed over this period. At −0.4 V_RHE_ Co_metal_ displays both the highest TOF but also the strongest binding toward *OH out of all the screened surfaces, sitting closer to the apex of a commonly employed volcano plot. All the other surfaces sit therefore in the weaker binding side. We would expect metal sheets that strongly bind oxygen species such as Fe, Mo or W to be limited by the release of NO_x_.^[^ ^84^ ^]^ We have additionally computed the NH_3_ adsorption energies to correlate it with the catalytic activity. However, our results indicate that OH binding energy exhibits a stronger correlation with the observed activity than NH_3_ adsorption energy (Figure S22, Supporting Information). This indicates that the intermediates binding via oxygen atoms such as NO_3_^−^ and NO_2_^−^ are the key species governing the reaction kinetics, which is consistent with previous reports.^[^ ^14^ ^]^

### 2.4 Energy Efficiency

**Figure 7:**
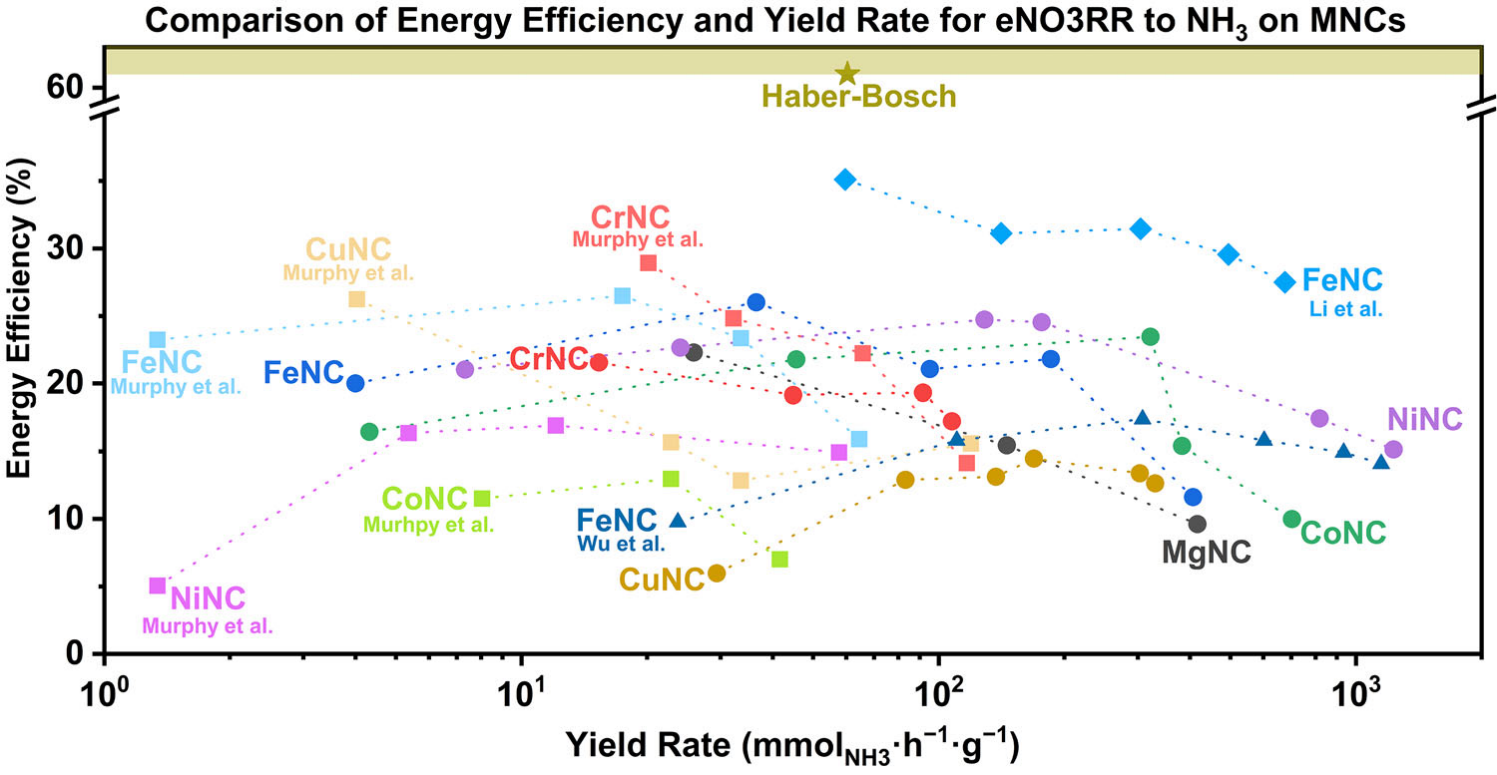
EE and ${\mathrm{YR}}_{{\mathrm{NH}}_{3}}$ of all MNCs investigated in this study after 1‐h electrolysis at the defined potential (0.1 mol·L^−1^ NaOH + 0.5 mol·L^−1^ NaNO_3_ with Ar 99.999% as purging gas in a H‐type cell with a graphite bar as CE and a Fumasep membrane to separate the compartments) and literature SACs materials. Reference MNC data from Murphy et al. (squares),^[^ ^14^ ^]^ Li et al. (diamonds)^[^ ^45^ ^]^ and Wu et al. (triangles).^[^ ^44^ ^]^ Haber‐Bosch values were obtained from Li et al.^[^ ^45^ ^]^ The YR values here were normalized by mass of catalysts. Only the mean value of EE and YR are being shown here. Individual plots for each material are provided in Figures S23 and S24 (Supporting Information) for clarity. Overpotential for counter reaction (oxygen evolution) was assumed to be zero for EE calculation (see Supplementary Information).

The energy efficiency (EE) of electrochemical processes is important for practical viability. The relationship between EE and $YR_{NH_{3}}$ is presented in **Figure** **7**. Among the evaluated materials, MgNC and CrNC exhibit limited variation in both efficiency and yield, with CrNC maintaining a narrow range of performance, similarly with Murphy et al.’s work,^[^ ^14^ ^]^ and MgNC showing a gradual decline in efficiency. FeNC achieves the highest EE of 26.0 ± 1.1% at –0.3 V_RHE_, despite showing a relatively low NH_3_ YR, 18.3 ± 0.3 µmol·h^−1^·${\mathrm{cm}}_{\mathrm{geo}}^{-2}$, compared to the other materials in this study. Nonetheless, the performance is comparable to FeNC reported by Li et al. (35.1% EE and 14.3 µmol·h^−1^·cm^−2^).^[^ ^45^ ^]^ In their work, Li and coworkers^[^ ^45^ ^]^ observed that for their FeNC, EE decreases with increasingly negative potentials, while ${\mathrm{YR}}_{{\mathrm{NH}}_{3}}$ increases, a trend mirrored in our findings. This behavior is likely attributable to the onset of competing side reactions at the cathode under more reducing conditions. At –0.6 V_RHE_ our more negative potential the FeNC achieved an EE of 11.6 ± 0.4%, while Li's FeNC achieved 27.5% at –0.7 V_RHE_. Wu et al. achieve in their work an EE ≈9‐18% between –0.5 to –0.85 V_RHE_ at pH ≈6_,_^[^ ^44^ ^]^ while for Murphy et al.’s work values between 15‐27% were achieved,^[^ ^14^ ^]^ showing that FeNCs tend to have an EE below 40%, aligning with our observations. CoNC displays a more variable trend, with EE increasing from –0.1 V_RHE_ until peaking at an EE of 23.5 ± 1.8%, with an ${\mathrm{YR}}_{{\mathrm{NH}}_{3}}$ of 160.9 ± 38.8 µmol·h^−1^·${\mathrm{cm}}_{\mathrm{geo}}^{-2}$ at –0.4 V_RHE_, followed by a decline in EE at –0.5 and –0.6 V_RHE_. This behavior contrasts with findings by Murphy et al.^[^ ^14^ ^]^ (more details in Table S2, Supporting Information), with values below 15%, but they do align with our SXRF (Figure 4) and DFT results (Figure 6), where increasing metal aggregation enhances TOF and ${\mathrm{YR}}_{{\mathrm{NH}}_{3}}$, but lowers EE due to side reactions occurring at different active sites. NiNC stands out, showing high EE at –0.1 V_RHE_ (21.0 ± 2.9%), that increases until –0.4 V_RHE_, then declines as ${\mathrm{YR}}_{{\mathrm{NH}}_{3}}$ rises at more cathodic potentials, also showing a contrast with Murphy et al.’s results (see Table S2, Supporting Information). This inverse correlation indicates a trade‐off between EE and YR at higher driving forces. NiNC offers the most balanced performance, maintaining EEs between 17.4 ± 3.7% and 15.1 ± 1.4%, while achieving high ${\mathrm{YR}}_{{\mathrm{NH}}_{3}}$ from 409.2 ± 160.8 to over 615.7 ± 176.5 µmol·h^−1^·${\mathrm{cm}}_{\mathrm{geo}}^{-2}$, closely followed by CoNC, which also exhibits favorable performance under similar conditions. CuNC has an EE ≈5‐15%, while Murphy et al.^[^ ^14^ ^]^ achieved an EE of 26% for their CuNC, but it also achieves ${\mathrm{YR}}_{{\mathrm{NH}}_{3}}$ above 100 µmol·h^−1^·${\mathrm{cm}}_{\mathrm{geo}}^{-2}$ at more reducing potentials.

When comparing the EE achieved in this work with some non‐SAC materials, it is observed that some high‐entropy alloys,^[^ ^85^ ^]^ can achieve up to 15 to 25% of EE, but with very low YR, but some other materials like CuCo aerogel,^[^ ^86^ ^]^ or spin polarized Fe_1_‐Ti,^[^ ^87^ ^]^ and others,^[^ ^88^, ^89^ ^]^ can achieve high EE and high YR (Figure S25 and Table S2, Supporting Information). Nevertheless, the field has not approached EE values achieved by the heavily established Haber‐Bosch process (≈61%),^[^ ^45^, ^90^ ^]^ therefore it becomes evident that there is still a significant gap to bridge in order to reach and maintain such levels of efficiency over extended periods, which could be breached via new reactor designs.^[^ ^91^ ^]^ Still, eNO_3_RR would reduce some processing costs when considering monetized environmental remediation. Nevertheless, when benchmarked against various MNCs and other materials reported in the literature for the eNO_3_RR (see Figure S25 and Table S2, Supporting Information), our materials—particularly NiNC and CoNC—demonstrate a promising balance between EE and $YR_{NH_{3}}$.

## 3 Conclusion 

This study reports on the electrochemical performance and in situ structural evolution of atomically dispersed MNCs (M = Cr, Fe, Co, Ni, and Cu). Results from 1‐h electrolysis in 0.1 mol·L^−1^ NaOH + 0.5 mol·L^−1^ NaNO_3_ indicates that all electrocatalysts exhibit above 50% $FE_{NH_{3}}$, except for CuNC which showed selectivity toward nitrite. On the other hand, although MgNC, CrNC and FeNC showed high $FE_{NH_{3}}$, the corresponding $YR_{NH_{3}}$ was considerable low compared the others (below 200 *µ*mol·h^−1^·${\mathrm{cm}}_{\mathrm{geo}}^{-2}$). NiNC stood out as the most selective and efficient electrocatalyst, with 78.0 ± 2.9% $FE_{NH_{3}}$ at −0.4 V_RHE_ and EE above 20% from −0.1 V_RHE_ to −0.4 V_RHE_ and ammonia production of 615.7 ± 176.5 *µ*mol·h^−1^·${\mathrm{cm}}_{\mathrm{geo}}^{-2}$ at −0.6 V_RHE_. CoNC was also highly promising with $FE_{NH_{3}}$ of 74.0 ± 4.6% at −0.4 V_RHE_, 351.4 ± 32.4 *µ*mol·h^−1^·${\mathrm{cm}}_{\mathrm{geo}}^{-2}$ at −0.6 V_RHE_, and EE above 20% between −0.3 and −0.4 V_RHE_. A minimum TOF (based on ICP‐MS) of 2.5·10^−15^ $\mu \mathrm{mo}l_{NH_{3}}$·h^−1^·site^−1^ at −0.4 V_RHE_ was achieved by CoNC. The reason for such a high TOF was explained by the structure‐activity plot of OH binding energy with experimental TOF, showing that structural transformation occurs from CoNC SAC to Co_metal_ below −0.2 V_RHE_. In situ SXRF maps of CoNC starting at OCP (0.15 V_RHE_) and reducing the cathodic potential from 0.0 V_RHE_ to −0.6 V_RHE_ at 100 mV steps also showed the clustering of Co. The formation of metal agglomerates in NiNC was also observed by in situ SXRF from OCP to 0.0 V_RHE_ and down to −0.6 V_RHE_. This was also confirmed from the structure‐activity trend, where NiNC SAC is weakly binding to OH, while the high TOF observed arises from the stronger OH binding of Ni_metal_. This work therefore elucidates the sensitive potential‐dependent nature of MNCs and simultaneously offers a clear guiding principle for producing intrinsically active eNO_3_RR catalysts based on their OH binding energy and in situ structural evolution.

## Conflict of Interest

The authors declare no conflict of interest.

## Author Contributions

D.S.B. and A.P. contributed equally to the manuscript. D.S.B. conducted the electrochemical and SXRF experiments, analyzed data, and wrote the original draft of the manuscript. A.P. conducted the synthesis and characterization of the materials, discussed and analyzed the theoretical calculations, and wrote the original draft of the manuscript. R.M. and A. B. conducted, discussed, and analyzed the theoretical calculations. J. B. conducted the synthesis and characterization of the materials and conceptualized the experiments. I.T.N. conducted and discussed the SXRF experiments. T.M.M. and M.G.W. contributed to electrochemical and SXRF experiments. I.E.L.S., M.M.T. provided funding and supervision. R.N. conceptualized and supervised the project. All authors provided critical feedback and helped shape the research, analysis, and manuscript.

## Supporting information



Supporting Information

## Data Availability

The data that support the findings of this study are available from the corresponding author upon reasonable request.
