# Supplementary material for: In Situ Structural Evolution and Activity Descriptor of Atomically Dispersed Catalysts During Nitrate Electroreduction
Source: Adv Sci (Weinh). 2025 Aug 22;12(39):e10282. doi: 10.1002/advs.202510282 (PMC12533412; doi:10.1002/advs.202510282)
Supplement: Supplementary file 1 — Supporting Information [file ADVS-12-e10282-s001.docx]

**Supplementary Information**

***In Situ* Structural Evolution and Activity Descriptor of Atomically Dispersed Catalysts During Nitrate Electroreduction**
Daniel S. Braga^a#^, Angus Pedersen^b,c#^, Mohd Riyaz^d^, Jesús Barrio^b,*^, Alexander Bagger^d^, Itamar T. Neckel^e^, Thiago M. Mariano^a,f^, Manuel E. G. Winkler^a,f^, Ifan E. L. Stephens^g^, Maria-Magdalena Titirici^b,h*^, Raphael Nagao^a,f,*^

^a^ Institute of Chemistry, University of Campinas, Campinas, SP 13083-862, Brazil

^b^ Department of Chemical Engineering, Imperial College London, London SW7 2AZ, United Kingdom

^c^ Division 3.6, Electrochemical Energy Materials, Bundesanstalt für Materialprüfung und -forschung (BAM), Unter den Eichen 44-46, 12203, Berlin, Germany.

^d^ Department of Physics, Technical University of Denmark, Kongens Lyngby 2800, Denmark

^e^ Brazilian Synchrotron Light Laboratory, Brazilian Center for Research in Energy and Materials, Campinas, SP 13083-100, Brazil

^f^ Center for Innovation on New Energies, University of Campinas, Campinas, SP 13083-084, Brazil

^g^ Department of Materials, Imperial College London, London SW7 2AZ, United Kingdom

^h^ Advanced Institute for Materials Research (WPI-AIMR), Tohoku University, 2-1-1 Katahira, Aobaku, Sendai, Miyagi, 980-8577, Japan.

[*j.barrio-hermida@imperial.ac.uk](mailto:*j.barrio-hermida@imperial.ac.uk), [m.titirici@imperial.ac.uk](mailto:m.titirici@imperial.ac.uk) and [nagao@unicamp.br](mailto:nagao@unicamp.br)

^#^These authors contributed to the manuscript equally.

**Experimental section**

**Synthesis**

Porous nitrogen doped carbon materials are prepared by high temperature pyrolysis of a mixture comprising 2,4,6-Triaminopyrimidine (TAP) and MgCl_2_^.^6H_2_O in 1:8 ratio. The mixture is achieved by grinding with a pestle and a mortar TAP and MgCl_2_^.^6H_2_O, which is then transferred to a ceramic crucible and pyrolyzed at 900 °C for 1-h at a heating rate of 5 °C^.^min^−1^ in N_2_ atmosphere. The resulting material is collected, washed with 45 mL of 2 mol·L^−1^ HCl overnight to eliminate remaining MgCl_2_ and MgO, filtered and washed thoroughly with DI water. Single atoms are coordinated within nitrogen doped carbon via low-temperature methanol reflux. Namely, 60 mg of porous nitrogen-doped carbon derived from TAP are dispersed in 75 mL of MeOH in a round bottom flask and subsequently 75 mL of a 25·10^−3^ mol·L^−1^ solution of a metal chloride is added (FeCl_2_, NiCl_2_·6H_2_O, CoCl_2_·6H_2_O, CrCl_3_·6H_2_O or CuCl_2_·2H_2_O). The resulting solution is then heated under reflux at 90 °C for 24 h, and the solid is collected by filtration. The solid is finally washed overnight with 0.5 mol·L^−1^ H_2_SO_4_ to remove metallic aggregates, filtered, washed thoroughly with DI water and dried at 80 °C in an oven.

**Characterization**

**Time-of-flight Secondary Ion Mass Spectrometry (ToF-SIMS):**

An ION-TOF V (IONTOF GmbH) was used CuNC and MgNC. Measurements used a 100 µm × 100 µm field of view using a Bi^3+^ primary ion beam with 25 keV and 0.4 pA beam current and 256 × 256 pixels. Analysis lasted 20 mins and images were rastered in sawtooth mode, with 100 µs cycle time (870 amu), employing a 5 µm spot size and flood gun. The sputter beam consisted of a 7.4 nA Ar beam (~1300 cluster size) at 10 keV rastered over 500 µm × 500 µm, with non-interlaced 1 sputter frames and 1 s pause, up to a dose density 10^15^ ions·cm^−2^. Negative spectra were calibrated to: C^–^, CH^–^, CH_2_^–^, C_3_^–^, C_4_^–^, C_5_^–^, C_6_^–^, C_7_^–^, C_8_^–^, C_9_^–^, with deviation < 50 ppm for all calibrations peaks in samples. Data analysis was performed using SurfaceLab 7 software, and normalization of peak intensity counts was computed based on the total ion count across the measured spectrum. All identified peaks in samples possessed < 100 ppm deviation from calculated atomic mass units and resolution < 3000 mass units. The measurement procedure was repeated at four different sample locations.

**X-ray Diffraction (XRD):**

Patterns (0.033 ^o^ scan step size) were obtained with a Malvern PANanalytical Aeris powder X-ray diffractometer using a Cu *K*α source operated at 45 kV and 30 mA from 5 to 60 ^o^ two-theta range.

**Inductively Coupled Plasma Mass Spectrometry (ICP-MS):**

Metal contents were obtained using an Agilent 7900 ICP-MS Spectrometer (Agilent Technologies). Powder samples were initially digested in aqua regia (25 v/v% HNO_3_ (69%, Certified AR, Eur.Ph., for analysis Fisher Chemical, Fisher Scientific and 75 v/v% HCl (37%, Certified AR, Eur.Ph., for analysis Fisher Chemical, Fisher Scientific) by employing a MARS 6 microwave at 1500 W for 15 min at 215 ^o^C. The resulting solutions were diluted to 2% HNO_3_ and measured against calibration standards containing metal concentrations of 0, 5, 50, 100, 200 and 500 ppb.

To convert from wt.% to at.% we neglect the present of N (which has a similar molecular weight to C):^[1]^

|  | ${at.\%}_{ICP-MS}=\frac{1}{\left[ 1+\frac{M_{\mathrm{metal}}\cdot(100\%-\mathrm{wt}_{ICP-MS})}{M_{\mathrm{carbon}}\cdot\mathrm{wt}_{ICP-MS}} \right]}$ | (S1) |
| --- | --- | --- |

where *wt._ICP-MS_* is metal (Mg, Fe, Co, Cu, Cr) wt.% determined by ICP-MS and *M_metal_* and *M_carbon_* is the molar mass of the metal and carbon (g mol^-1^), respectively.

**X-ray Photoelectron Spectroscopy (XPS):**

XPS data were obtained with a Thermo fisher K-alpha system with a monochromated, micro-focused Al Kα source, 400 μm spot size, and the spectra were analyzed in the Thermo Scientific Avantage software 5.9931.

**Electrochemical measurements**

All electrochemical procedures were carried out in potentiostatic mode using the Autolab PGSTAT302N workstation at room temperature under Argon (White Martins, 99.999% gas atmosphere, with prior bubbling for 10-15 minutes). A 3-electrode cell was used with a graphite bar as the auxiliary electrode, a reversible hydrogen electrode (RHE) in 0.1 mol·L^−1^ NaOH as the reference, carbon cloth (CeTech Carbon Cloth W1S1010) with electrocatalyst deposited by drop casting as the working electrode (0.5 mg·cm^−2^) with a geometric area of 0.25 cm^2^, and a Fuel Cell Store Fumasep (FAB-PK-130) membrane to separate the cell compartments. The RE–WE and WE–CE distances were 1.0 cm and 7.5 cm, respectively; the cell height was 11.0 cm, compartment separation was 5.5 cm, and each compartment had a 2.0 cm diameter. The total cell width was 11.0 cm with the reference electrode compartment and 9.5 cm without it. A 3D schematic model of electrochemical cell can be found in Figure S8.

The suspensions of the materials were prepared at a concentration of 10 mg·mL^−1^ in 2-propanol and 5% Nafion in a ratio of 96:4 (2-propanol:Nafion) as a solvent and homogenized by ultrasound in an ultrasonic bath for 30 minutes after preparation and between 5 and 10 minutes before each experiment. For all metals, the following procedure was adopted: catalyst inks were prepared and stored at 10 °C, and electrochemical measurements were initiated the following day. The first potential step (−0.1 V_RHE_) was performed on day 1, and subsequent measurements (up to −0.6 V_RHE_) were completed within a maximum of 6 days after ink preparation. Prior to use, the carbon cloth was cleaned in an ultrasonic bath for 10 minutes sequentially in acetone, ethanol, and Milli-Q water, then dried in an oven at approximately 70 °C.

**Impedance (EIS)**

All the electrochemical tests were preceded by an electrochemical impedance spectroscopy experiment to determine the uncompensated resistance (R_u_). EIS was carried out in potentiostatic mode using a frequency range of 1·10^5^ Hz to 1·10^−1^ Hz, with a perturbation of 10 mV_RMS_ applied around the open circuit potential (OCP) to maintain proximity to equilibrium. R_u_ was determined by the value closest to intercepting the imaginary impedance axis on the Nyquist plot (not fitted with an equivalent circuit).

85% of R_u_ value was offset during the measure. For measurements in 0.1 mol·L^−1^ NaOH R_u_ was 30-40 Ω, for measurements in 0.1 mol·L^−1^ NaOH + 0.5 mol·L^−1^ NaNO_3_ R_u_ was 10-20 Ω.

**Cyclic voltammetry (CV)**

Before each chronoamperometry experiment, 5 voltammetry cycles—in 0.1 mol·L^−1^ NaOH and 5 cycles in 0.1 mol·L^−1^ NaOH + 0.5 mol·L^−1^ NaNO_3_—were carried out to check the reproducibility of the ink deposition and determine the onset potential. The reaction onset potential was determined to be the potential at which a current density of 0.4 mA·cm^−2^ was reached. CV was carried out in potentiostatic mode at 20 mV·s^−1^ from +0.3 to −0.6 V_RHE_.

**Chronoamperometry (CA)**

**Chronoamperometry** was performed for one hour in 0.1 mol·L^−1^ NaOH + 0.5 mol·L^−1^ NaNO_3_ at room temperature under inert gas atmosphere (with prior bubbling for 10-15 minutes), with stirring at ~700 rpm. Each CA experiment was measured at least twice.

**Nitrite was quantified** using absorption spectroscopy in the visible region (~543 nm) using the Griess reaction, following the procedure of an external calibration curve between the concentrations of 0.00 and 0.16 ppm of NO_2_^−^. The Griess reagent is prepared with 1 gram of sulfanilamide, 0.1 gram of N-(1-Naphthyl)ethylenediamine-dihydro-chloride and 10 mL of H_3_PO_4_ ≥ 85%, finally the volume is adjusted to 100 mL using milli-q water. All samples were adjusted to fall within the range of the standard curve. The Griess reagent was stored – in an amber bottle, in a fridge at 10 ºC, and wrapped in aluminum foil. – for a maximum of one month, and a new calibration curve was made whenever the reagent needed to be renewed.

**Ammonia was quantified** using absorption spectroscopy in the visible region (~690 nm) via the Berthelot reaction. The standard addition method was applied to correct for rotational matrix effects, using NH₃ concentrations ranging from 0.00 to 1.00 ppm. To account for translational matrix effects, a sample of the electrolyte prior to electrolysis was measured. The maximum absorbance from this baseline sample was then subtracted from the absorbance of all other samples. A Merck SpectroQuant (HC200877) ammonia quantification kit was used. All samples were adjusted to fall within the concentration range of the Merck kit (0.06 - 3.6 ppm of NH_3_). The limit of detection and limit of quantification are determined by the expression below:

|  | $LOD= \frac{3.3\cdot\sqrt{\frac{\sum\left( y-\hat{y} \right)^{2}}{n-2}}}{\frac{n\cdot(\sum x\cdot y)-(\sum x)\cdot(\sum y)}{n\cdot(\sum x^{2})-{(\sum x)}^{2}}}$ | (S2) |
| --- | --- | --- |
|  |  |  |
|  | $LOQ=10\cdot LOD$ | (S3) |

where LOD stands for limit of detection, LOQ for limit of quantification, n for the number of samples, x for the ammonia concentration, y for the absorbance values and $\hat{y}$ for the predicted absorbance values based on the linear regression model. Alternatively, any spreadsheet software should be capable of performing the LOD expression by doing $\frac{3.3\cdot STEYX(abs, conc)}{SLOPE(abs, conc)}$.

Any measure in ammonia determination by absorption spectroscopy method was set to zero (0) if the value determined was below LOD.

Quantification via absorption spectroscopy in the visible region was carried out using a spectrophotometer (Bel Photonics UV - M51) with a wavelength range between 190 and 1000 nm and absorbance between −0.3 and 3.0, using a 1 cm quartz cuvette. The equipment's baseline for all the samples was made with milli-Q water.

**Ammonia was also quantified via ^1^H NMR** to check the accuracy of quantification via UV/Vis, in addition to being measured with labelled nitrate (^15^NO_3_^−^) to identify possible presence of alternative sources of nitrogen in the electrolyte, following the procedure of internal standard method. The pH of the sample was adjusted to ~3 with H_2_SO_4_. Dimethyl sulfone was used as internal standard, with DMSO-*d_6_* to lock the signal and to reference chemical shift of other substances. A small modification of ZGESGP – to better adjust the measure in the equipment – was used to 1D water suppression. 64 scans (+4 dummy scans) with 30 seconds as relaxation delay. The formula to determine the concentration of ammonia (note that the formula provides mass of NH_4_^+^, since this is the analyte in the measure) via ^1^H NMR is presented below:

|  | $m[mg]=\frac{A[a.u.]\cdot N_{\mathrm{IS}}[a.u.]\cdot m_{\mathrm{IS}}[mg]\cdot MM[g\cdot\mathrm{mol}^{-1}] \cdot P_{\mathrm{IS}}[a.u.]}{N[a.u.]\cdot A_{\mathrm{IS}}[a.u.]\cdot MM_{\mathrm{IS}}[g\cdot\mathrm{mol}^{-1}]}$ | (S4) |
| --- | --- | --- |

where m stands for mass, A for area, N for number of hydrogen (6 for IS and 4 for NH_4_^+^), MM for molar mass and P for purity.

^1^H NMR measurements were carried out at Bruker Avance III 9.4T, 400 MHz.

**Faradaic efficiency (FE)** was determined using the area under the CA curve as the charge and the concentration of the product in ppm of the product, the formula is given below:

|  | $FE[\%]=\frac{x\left[ mg\cdot\text{L}^{-\text{1}} \right]\cdot V\left[ L \right]}{\mathrm{MM}_{x}\left[ mg\cdot\text{mol}^{-\text{1}} \right]}\cdot\frac{Z\cdot F\left[ C\cdot\text{mol}^{-\text{1}} \right]}{Q\left[ C \right]}\cdot100[\%]$ | (S5) |
| --- | --- | --- |
|  |  |  |

where x stands for concentration of the product (in ppm of the product), V for the total volume in cell chamber, MM for molar mass of the product, Z for the number of electrons involved in the step reaction (i.e. 8 for ammonia, 2 for nitrite, dimensionless), F for Faraday constant and Q for absolute charge.

**Yield rate (YR)** was determined using the concentration of the product in ppm of the product and total time of reaction, the formula is given below:

|  | $\mathrm{YR}\text{[}\text{µmol·cm}^{-\text{2}}\text{·}\text{h}^{-\text{1}}]= \frac{x\left[ mg\cdot\text{L}^{-\text{1}} \right]\cdot V\left[ L \right]}{\mathrm{MM}_{x}\left[ mg\cdot\text{mol}^{-\text{1}} \right]}\cdot\frac{1}{A\text{[}\text{cm}^{\text{2}}\text{]}\cdot h[hours]}\cdot\frac{{10}^{6}[\mu mol]}{1\text{[mol]}}$ | (S6) |
| --- | --- | --- |

where x stands for concentration of the product (in ppm of the product), V for the total volume in cell chamber, MM for molar mass of the product, A for the area (in this work geometric area was used) and h for the total time of reaction.

**Partial current density** was determined using FE, previously determined, and the absolute charge over the total reaction time in seconds, the formula is given below:

|  | $\text{J}_{\text{partial}}\text{[mA·}\text{cm}^{-\text{2}}\text{]=}\frac{\text{FE[\%]}}{\text{100[\%]}}\text{·}\frac{\text{Q[C]}}{\text{h[hours]·}\frac{\text{3600[secs]}}{\text{1[hours]}}}\text{·}\frac{\text{1}}{\text{A[}\text{cm}^{\text{2}}\text{]}}\text{·}\frac{\text{10}^{\text{3}}\text{[mA]}}{\text{1[A]}}$ | (S7) |
| --- | --- | --- |

where FE stands for faradaic efficiency, Q for absolute charge, h for total time of reaction and A for the area (in this work geometric area was used).

**Site density (SD_ICP_)** was derived from metal content from ICP-MS:

|  | $\text{SD}_{\text{ICP}}\text{[site·}\text{g}^{-\text{1}}\text{]=}\text{N}_{\text{A}}\text{[site·}\text{mol}^{-\text{1}}\text{]·}\frac{\text{ICP[wt.\%]}}{\text{100[wt.\%]·M[g·}\text{mol}^{-\text{1}}\text{]}}$ | (S8) |
| --- | --- | --- |

where N_A_ is Avogadro’s constant (6.023·10^23^ site·mol^−1^), ICP is the wt.% of the metal, M is the molar mass of the active metal element.

**Turnover frequency (TOF)** towards NH_3_ production was determined via:

|  | $TOF[{\mu\mathrm{mol}}_{\mathrm{NH}_{3}}\cdot\mathrm{site}^{-1}\cdot h^{-1}]=\frac{YR[{\mu mol\cdot cm}^{-2}\cdot h^{-1}]}{L[g_{\mathrm{MNC}}\cdot\mathrm{cm}^{-1}]\cdot\mathrm{SD}_{\mathrm{ICP}}[site\cdot g^{-1}]}$ | (S9) |
| --- | --- | --- |

where L is the catalyst loading (g_MNC_ cm^−2^).

**Energy Efficiency (EE)** for producing ammonia was determined using FE, previously determined, and assuming the overpotential of anodic electrode (the water oxidation) against RHE in pH 13 is zero, the formula is given below:

|  | $\text{EE[\%] = }\frac{\left( \text{Eº}_{\text{anode}}\text{[V] }-\text{Eº}_{\text{cathode}}\text{[V]} \right)}{\text{(}\text{Eº}_{\text{anode}}\text{[V]}-\text{ E[V])}}\cdot\text{FE[\%]}$ | (S10) |
| --- | --- | --- |

where FE stands for faradaic efficiency and E is the potential of the anode/cathode.

**Stability test (ST)**

The same electrochemical cell setup was used as in the standard experiments. For ammonia and nitrite quantification, the appropriate electrolyte volume was considered at each time point, accounting for the volume removed during hourly aliquot sampling.

**Synchrotron X-ray fluorescence (SXRF)**

The *in situ* SXRF experiments for CoNC and NiNC were conducted *in situ* at the CARNAÚBA beamline of the Brazilian Synchrotron Light Laboratory (LNLS) on the Sirius facility. For each measurement, in a 0.1 mol·L^−1^ NaOH + 0.5 mol·L^−1^ NaNO_3_ solution, the sample was polarized at the desired potential, followed by a 5-minute stabilization period before the spectrum was measured. The measurements were taken with a beam spot roughly 200 × 500 nm^2^. Two-dimensional XRF maps were collected scanning the sample about the beam over areas of 50 × 50 µm (pixel of 500 nm) and 10 × 10 µm (pixel of 100 nm). For both metals, we selected regions with strong metal intensity for resolvable measurements. The contrast shown on the maps corresponds to the concentration of cobalt and nickel, which were obtained selecting the emission energy on the SXRF sum spectrum (Kα line) for the elements—*e.g.* 6931 eV for cobalt and 7480 eV for nickel. The working electrode was a carbon cloth fixed in a glassy carbon using a very small drop of silver/silver chloride paste for screen printing, with a CoNC or NiNC catalyst loading of 0.5 mg·cm^−2^, the counter electrode was platinum ring and the reference electrode was an Ag/AgCl leak free that was calibrated using a RHE at pH 13 prior the *in situ* SXRF experiments. To ensure reliability, the reference electrode was also checked before and after each measurement, showing no potential drift. Additional information about the *in situ* cell can be found in Ref.^[2,3]^ A photography of the cell is shown in Figure S16.

The data was normalized using the min-max technique, as described below in Equation S11.

|  | $\mathrm{Intensity}\left( a.u. \right) = \frac{Intensity - \mathrm{Intensity}_{\min}}{\mathrm{Intensity}_{\max} - \mathrm{Intensity}_{\min}}$ | (S11) |
| --- | --- | --- |

where Intensity (a.u.) stands for the matrix of the normalized values for the SXRF map. Intensity_min_ and Intensity_max_ are the minimum and maximum values in the matrix of the raw map, respectively.

**Computational details**

The DFT calculations were performed by Vienna ab initio Simulation package (VASP) using spin-polarized density functional employing the revised-Perdew-Burke-Ernzerhof (RPBE) exchange-correlation functional with the projector augmented wave (PAW) pseudopotential method to describe interactions between the core and electrons.^[4–6]^ Besides, the DFT-D3 method of Grimme was used to accommodate long-range van der Waals interactions.^[7]^

The MNC model utilized in this study consists of a metal atom coordinated between pyrrolic-N and pyridinic-N. To validate the model, we evaluated the H-binding energy on the surfaces and compared it to the HER (Hydrogen Evolution Reaction) volcano plot^[8]^ with respect to the HER onset potential. The results, shown in Figure S20, indicate that early transition metals like Cr, Fe, and Co align well with the volcano plot, compared to Cu and Ni. Furthermore, the metallic form of Ni demonstrates a strong fit in the volcano plot, suggesting that the HER activity predominantly arises from the metal cluster.


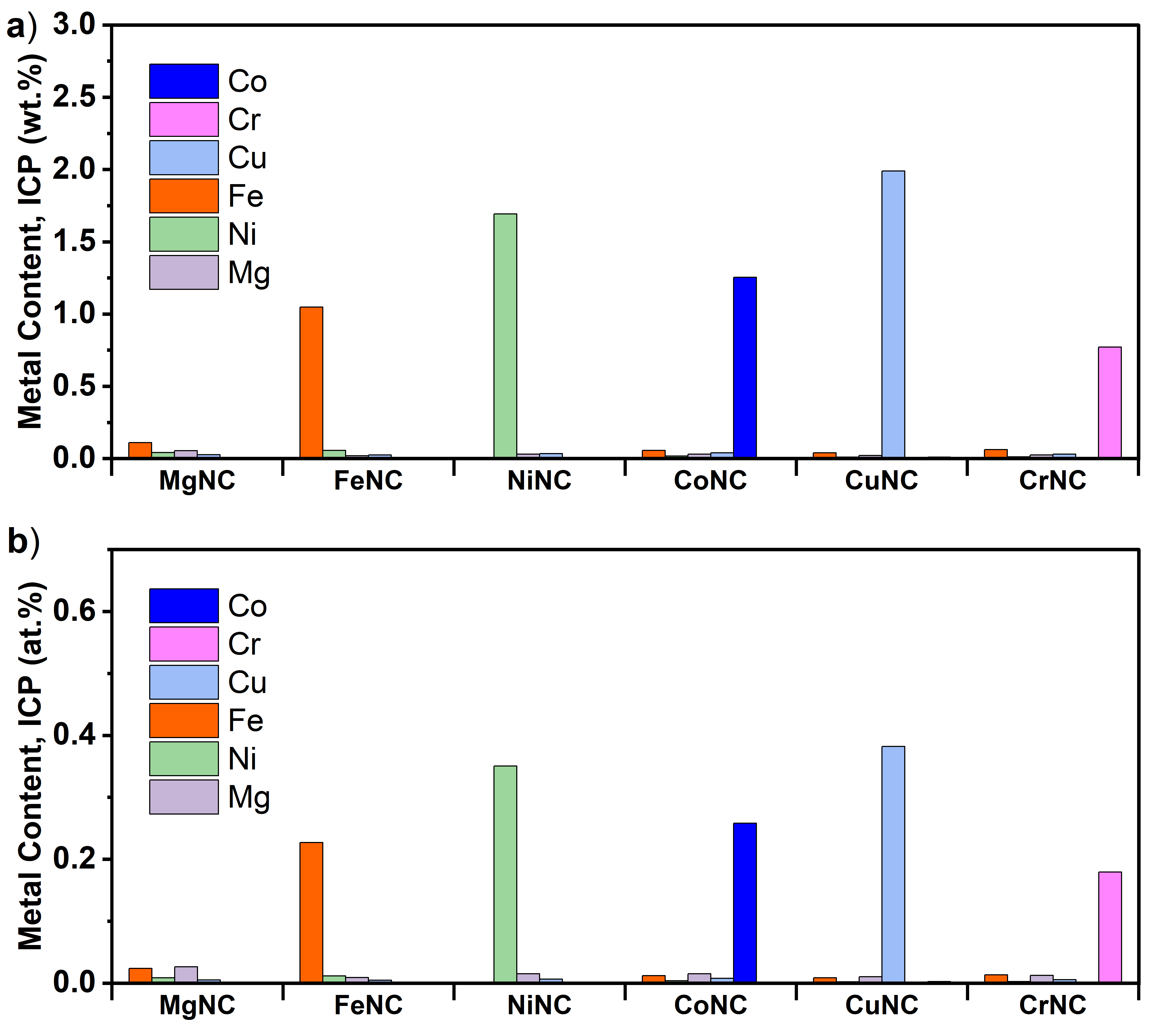


Figure S1 – ICP-MS of MNCs in terms of wt.% (a) at.% (b).


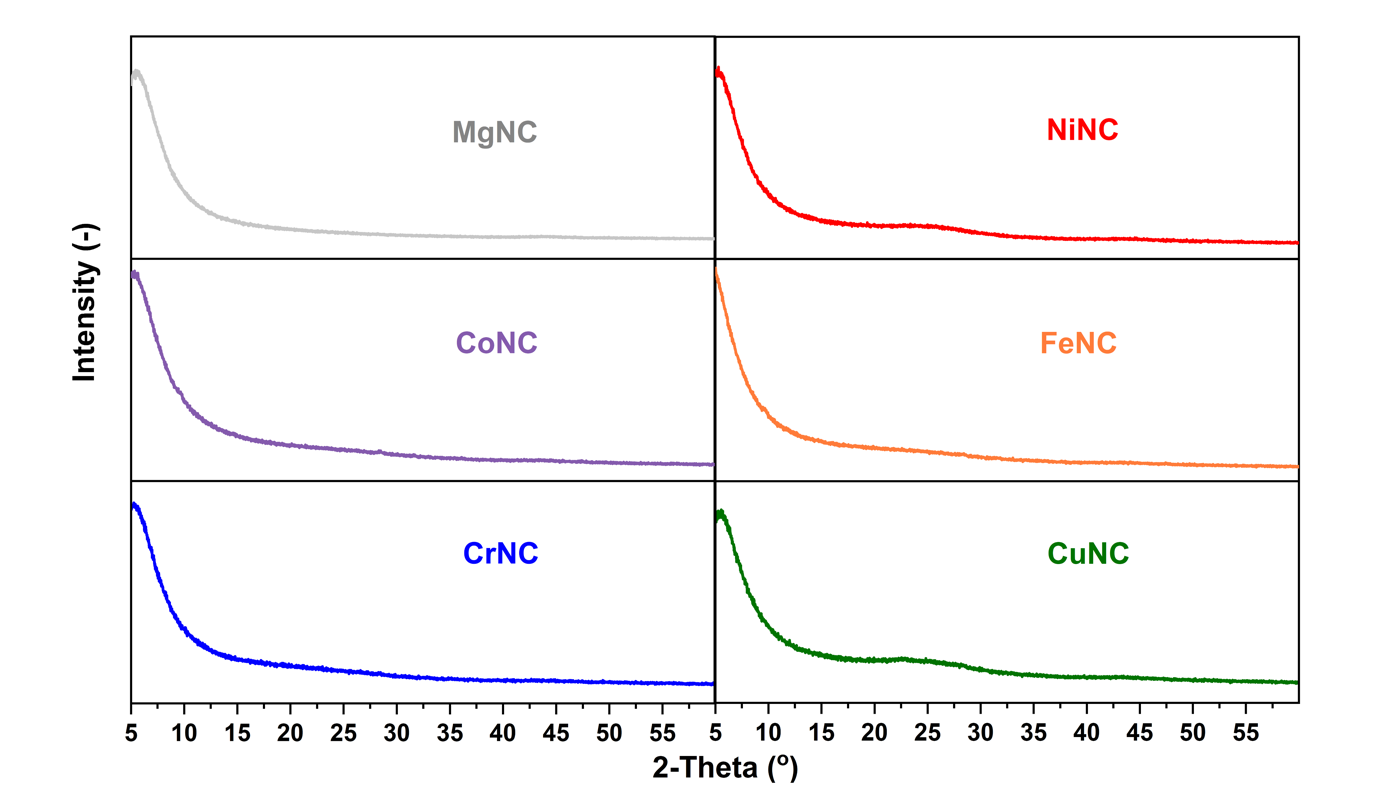


Figure S2 – X-ray diffraction patterns of the prepared MNC materials.


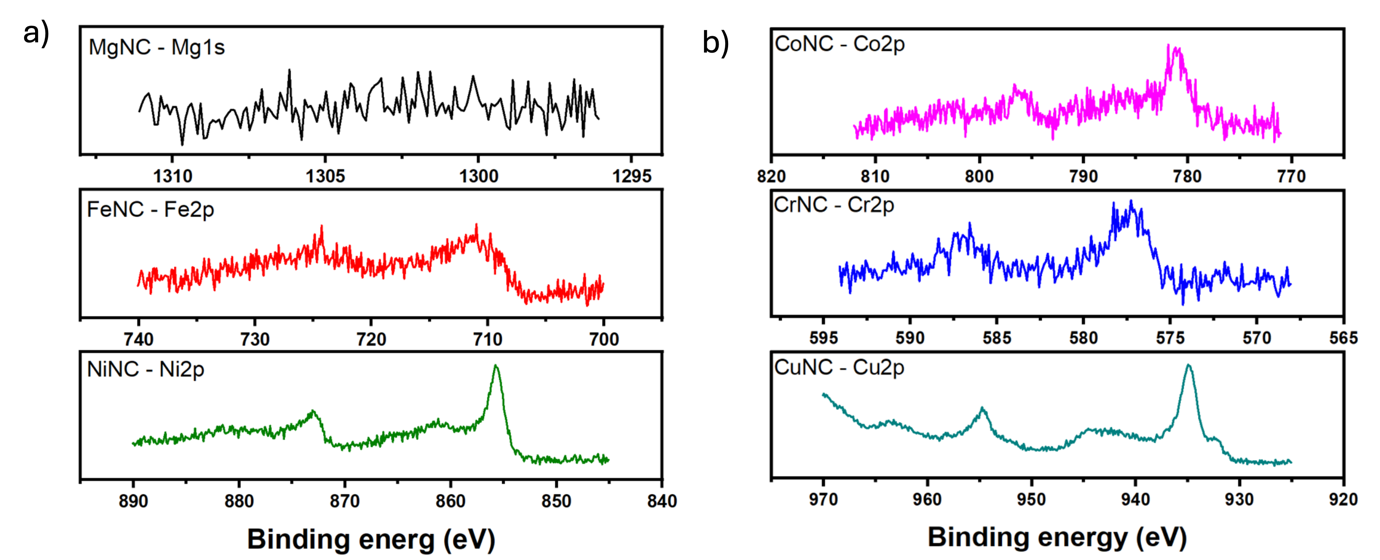


Figure S3 – XPS scan for the metals of interest in each of the MNCs.


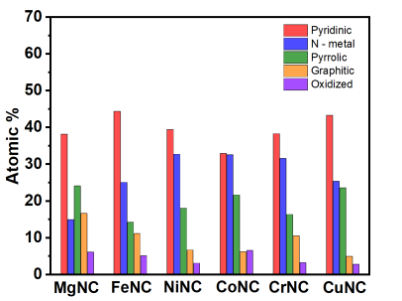


Figure S4 – Atomic percentage of the different nitrogen contributions obtained by XPS.

Table S1 – Binding energies (eV) of the nitrogen contributions observed by XPS.


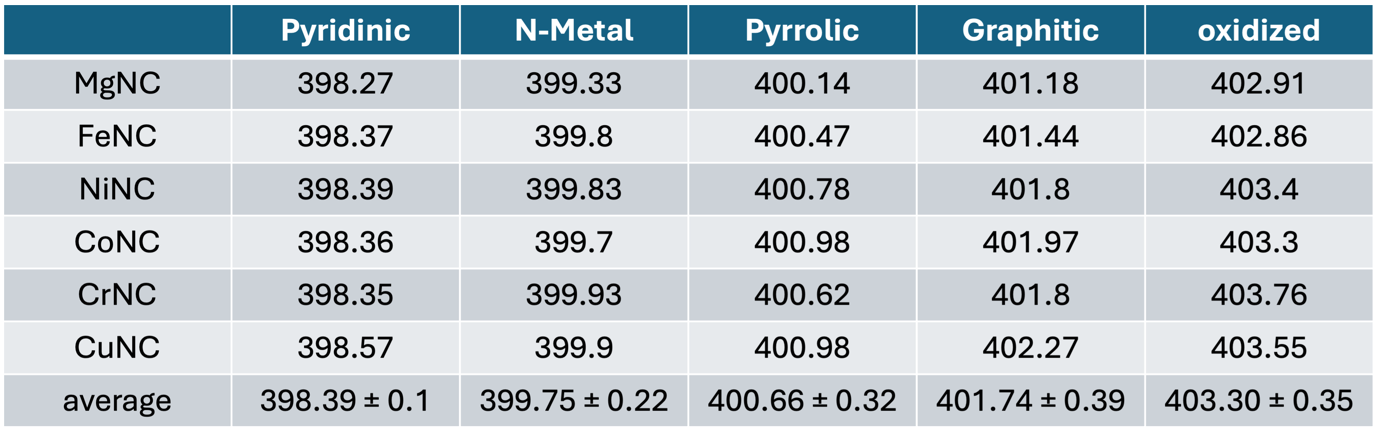


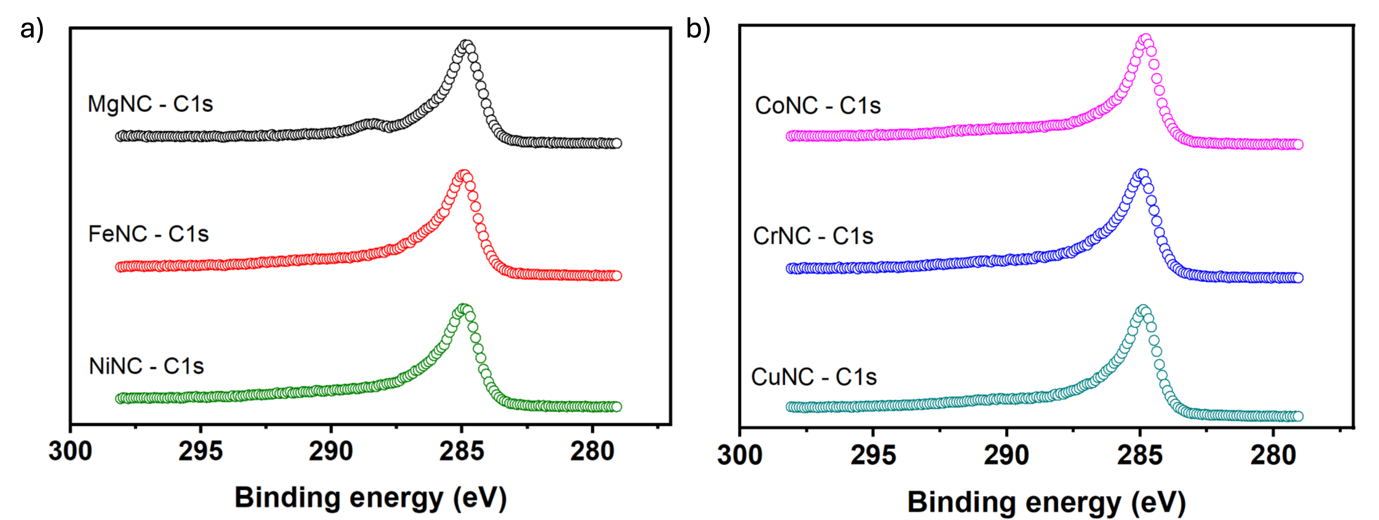


Figure S5 – C1s spectrum of the prepared MNCs.


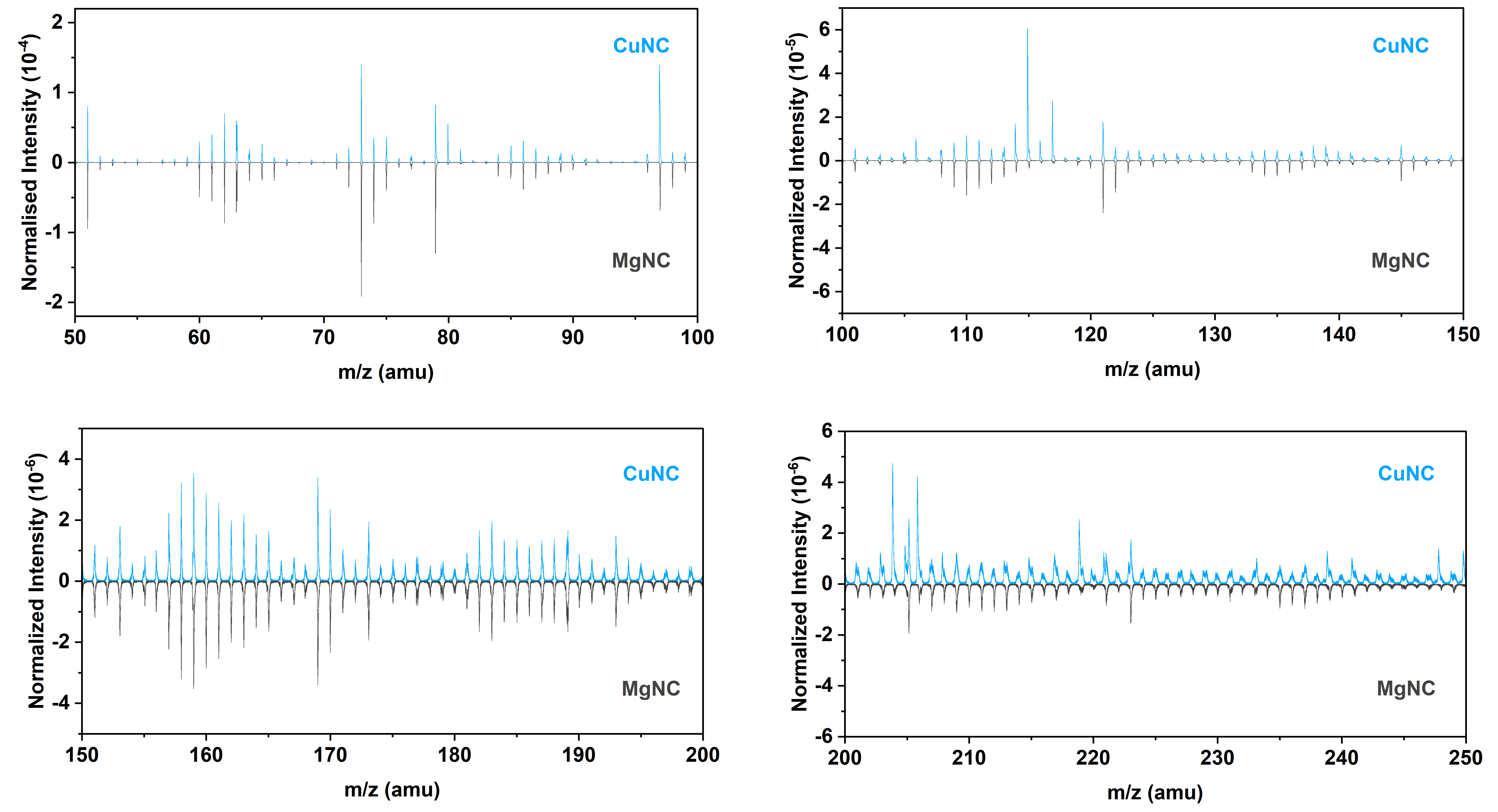


Figure S6 – ToF-SIMS comparison of CuNC and MgNC in negative polarity SIMS between 50-250 amu. A Bi^3+^ primary ion beam over 100 µm × 100 µm field of view with 25 keV and 0.5 pA beam current was used, while the sputter beam consisted of a 11 nA Ar beam (~1600 cluster size) at 10 keV rastered over 500 µm × 500 µm with non-interlaced 1 sputter frames and 1 s pause, up to a dose density 10^15^ ions·cm^−2^.

Figure S7 – Chemical mapping over 100 µm × 100 µm field of view for CuN_x_C_y_^−^ ion fragments and their counts compared to the total ions counts.


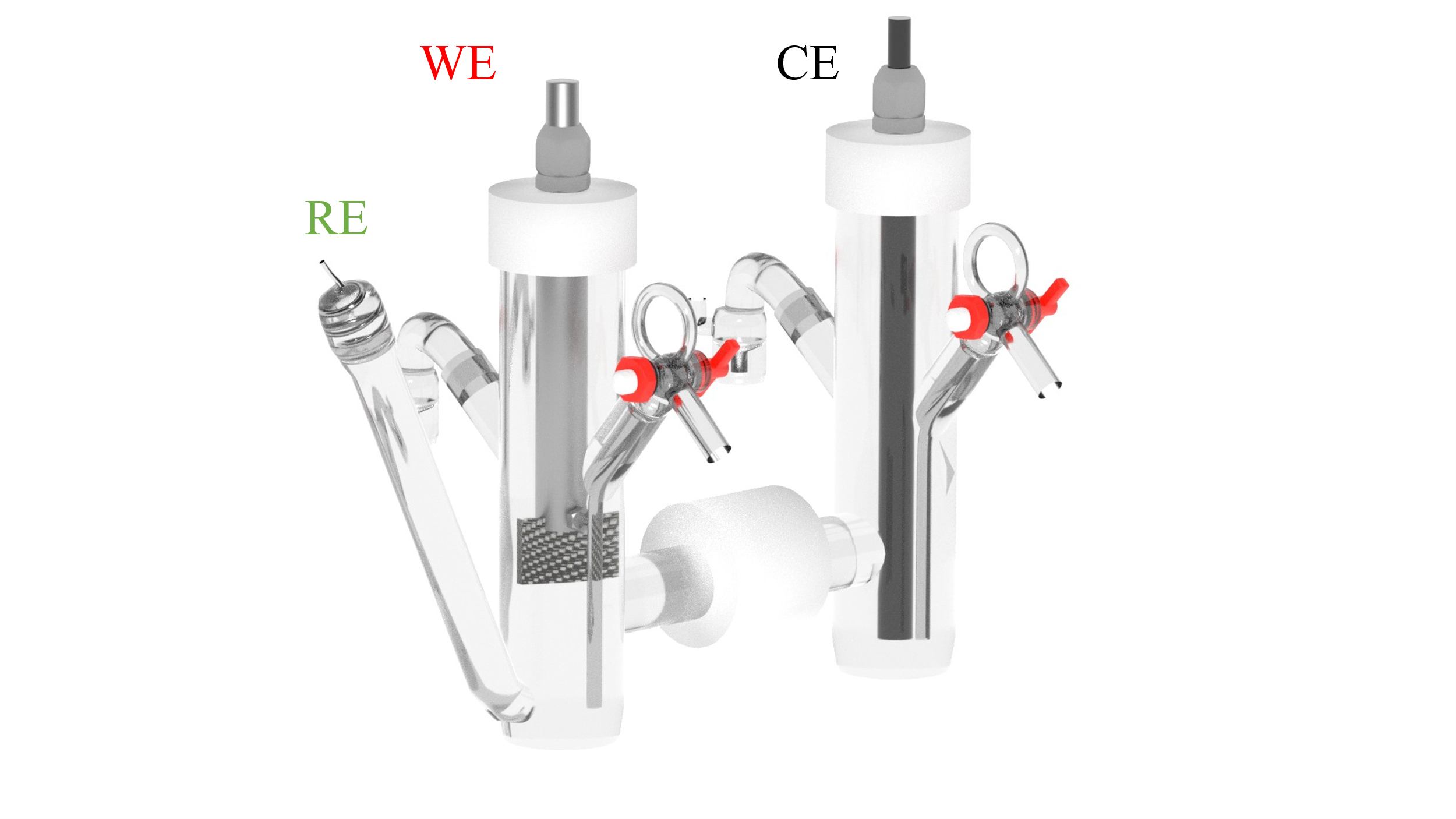


Figure S8 – 3D schematic model of the electrochemical cell. Distances: RE–WE = 1.0 cm, WE–CE = 7.5 cm, compartment separation = 5.5 cm, cell height = 11.0 cm, compartment diameter = 2.0 cm, cell width = 11.0 cm (with RE) / 9.5 cm (without RE).


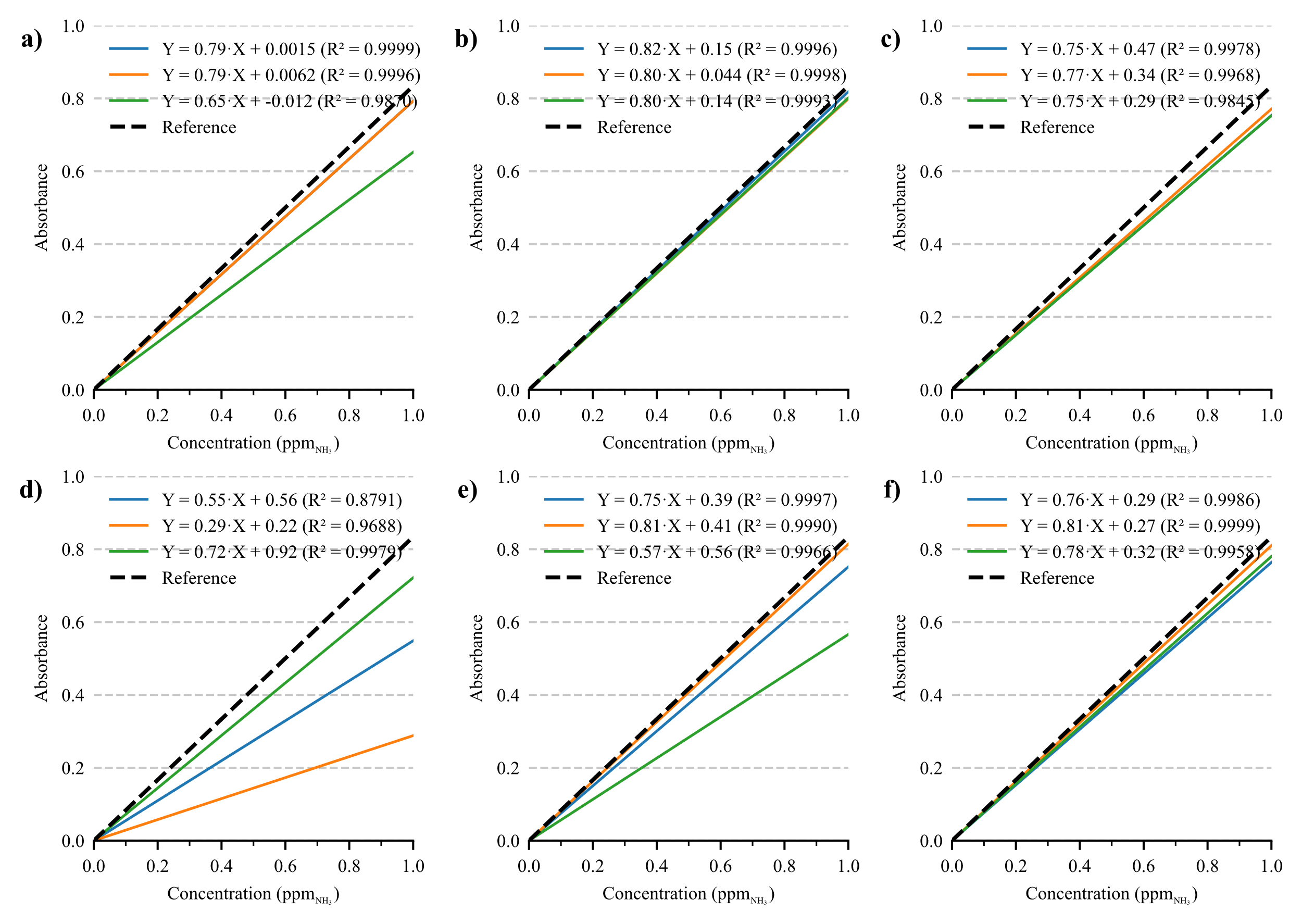


Figure S9 – Examples of ammonia standard addition method linear regression for CoNC in all six potentials studied in this work: −0.1 (a), −0.2 (b), −0.3 (c), −0.4 (d), −0.5 (e) and −0.6 V_−iR,RHE_ (f). All measurements were done in triplicate, with the dashed curve representing the standard curve from the Merck kit. The lines are plotted without linear coefficients to highlight differences in angular coefficients.


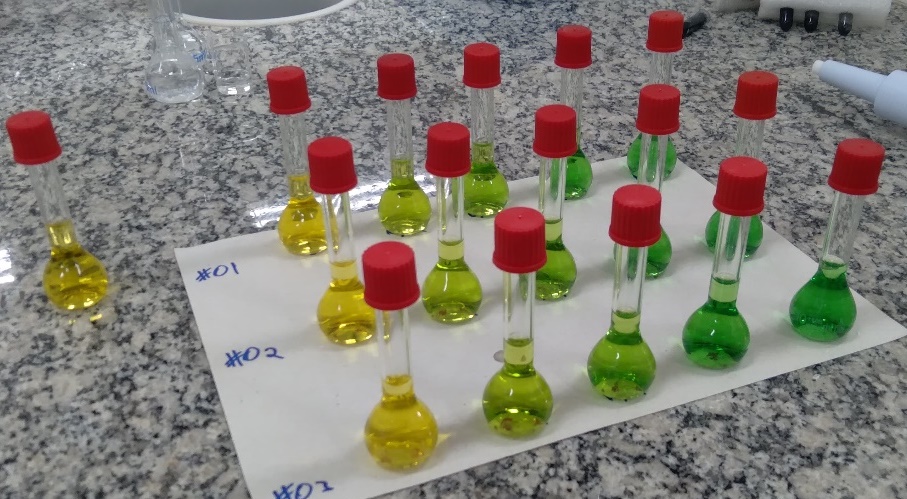


Figure S10 – Example of the preparation of curves using the standard addition method for a material in triplicate, with the flask on the left being the interferent (made only with the pre-electrolysis electrolyte). This is an experiment of a low potential where no ammonia was formed.


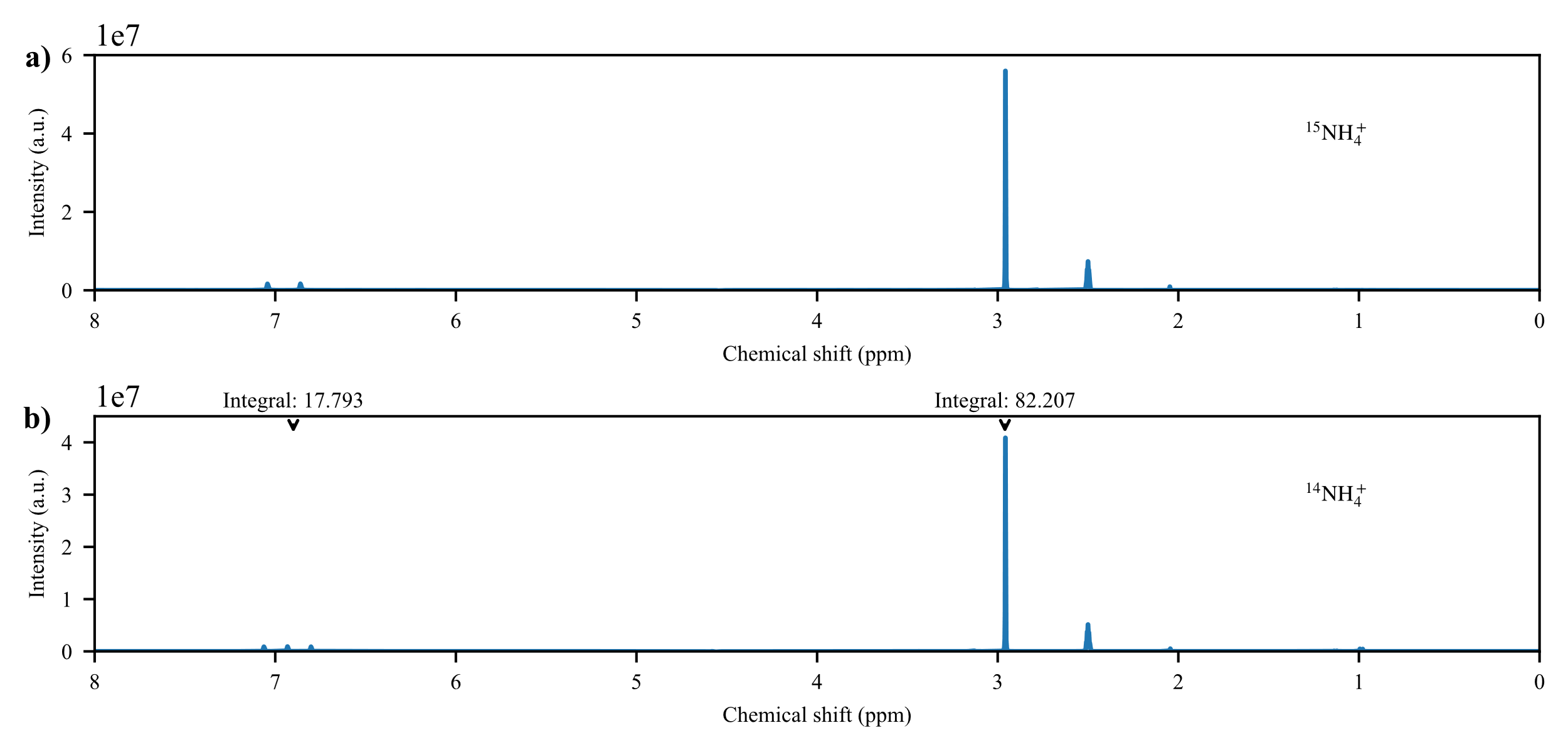


Figure S11 – ^1^H NMR with ^15^N-NH_4_^+^ (a) and ^14^N-NH_4_^+^ (b) referenced by DMSO-d_6_ at 2.5 ppm. NH_4_^+^ has a chemical shift around 6.9 ppm with two peaks when formed from ^15^NO_3_^−^, three peaks when formed from ^15^NO_3_^−^, DMSO_2_ used as internal standard has chemical shift around 2.9 ppm.


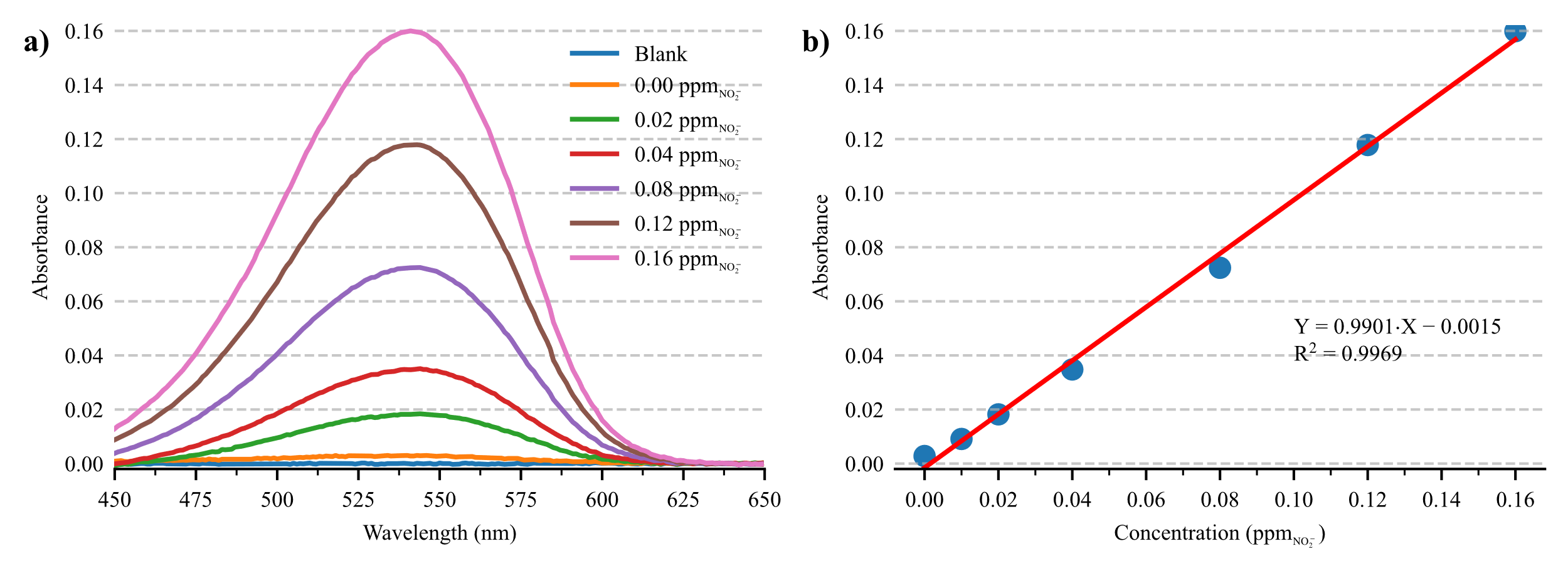


Figure S12 – Example of nitrite external calibration curve made with six concentrations between 0.00 and 0.16 ppm of nitrite (a), where a blank sample (made with milli-Q water) was run to compare with the 0.00 ppm of nitrite and linear regression of the curve (b).


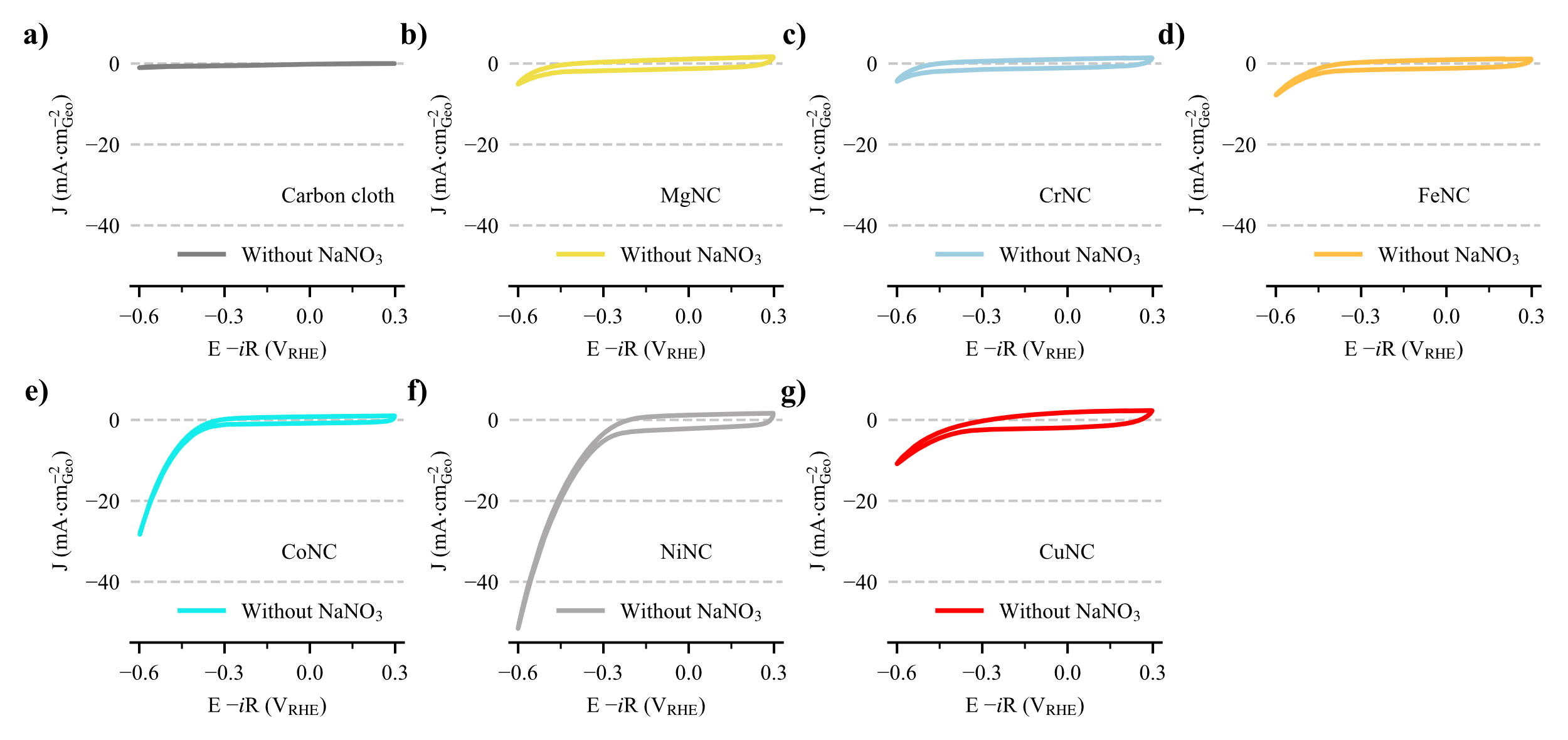


Figure S13 – 5^th^ CV at 20 mV·s^−1^, from +0.3 to −0.6 V_−iR,RHE_, in 0.1 mol·L^−1^ NaOH with Carbon cloth (a), MgNC (b), CrNC (c), FeNC (d), CoNC (e), NiNC (f) and CuNC (g).


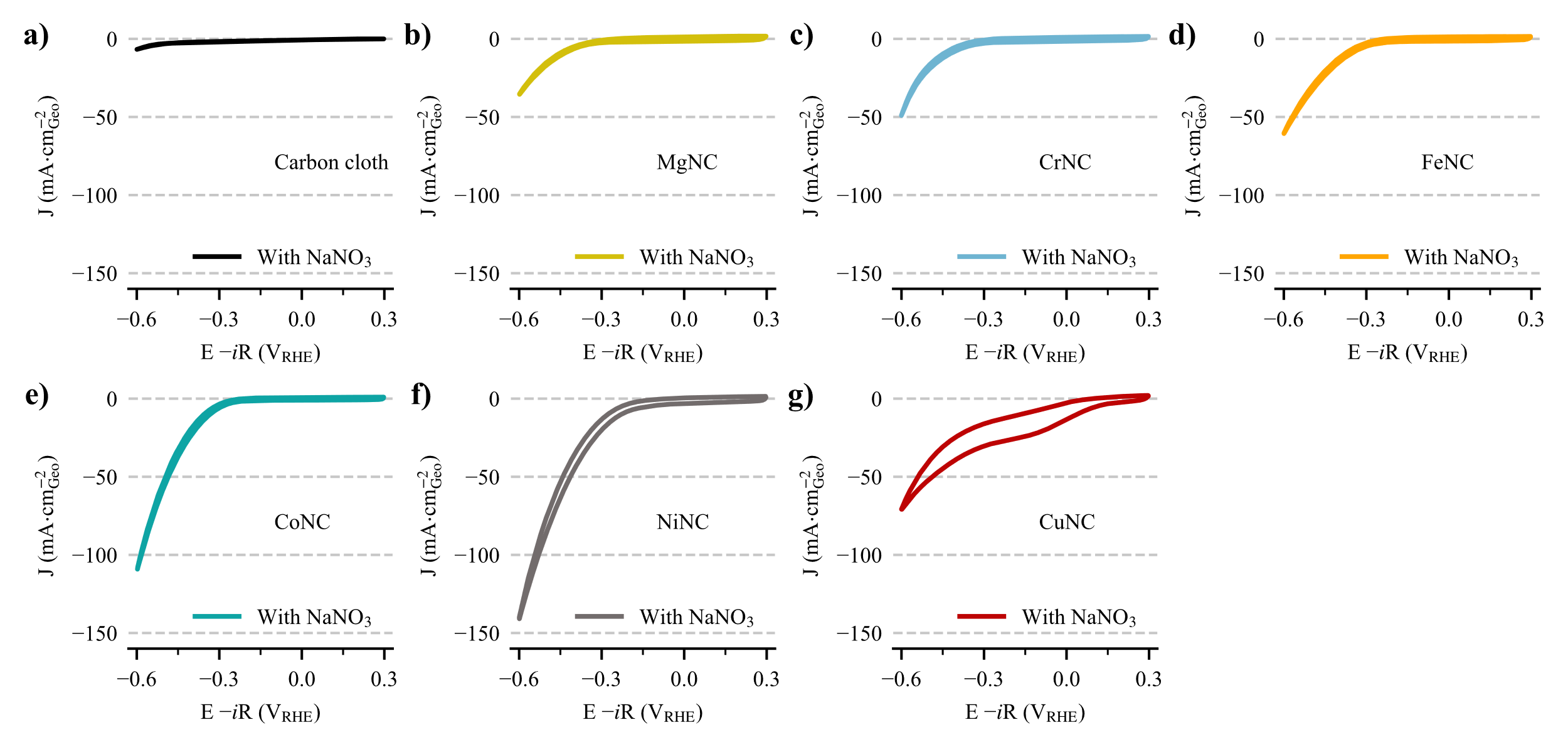


Figure S14 – 5^th^ CV at 20 mV·s^−1^, from +0.3 to −0.6 V_−iR,RHE_, in 0.1 mol·L^−1^ NaOH + 0.5 mol·L^−1^ NaNO_3_ with Carbon cloth (a), MgNC (b), CrNC (c), FeNC (d), CoNC (e), NiNC (f) and CuNC (g).


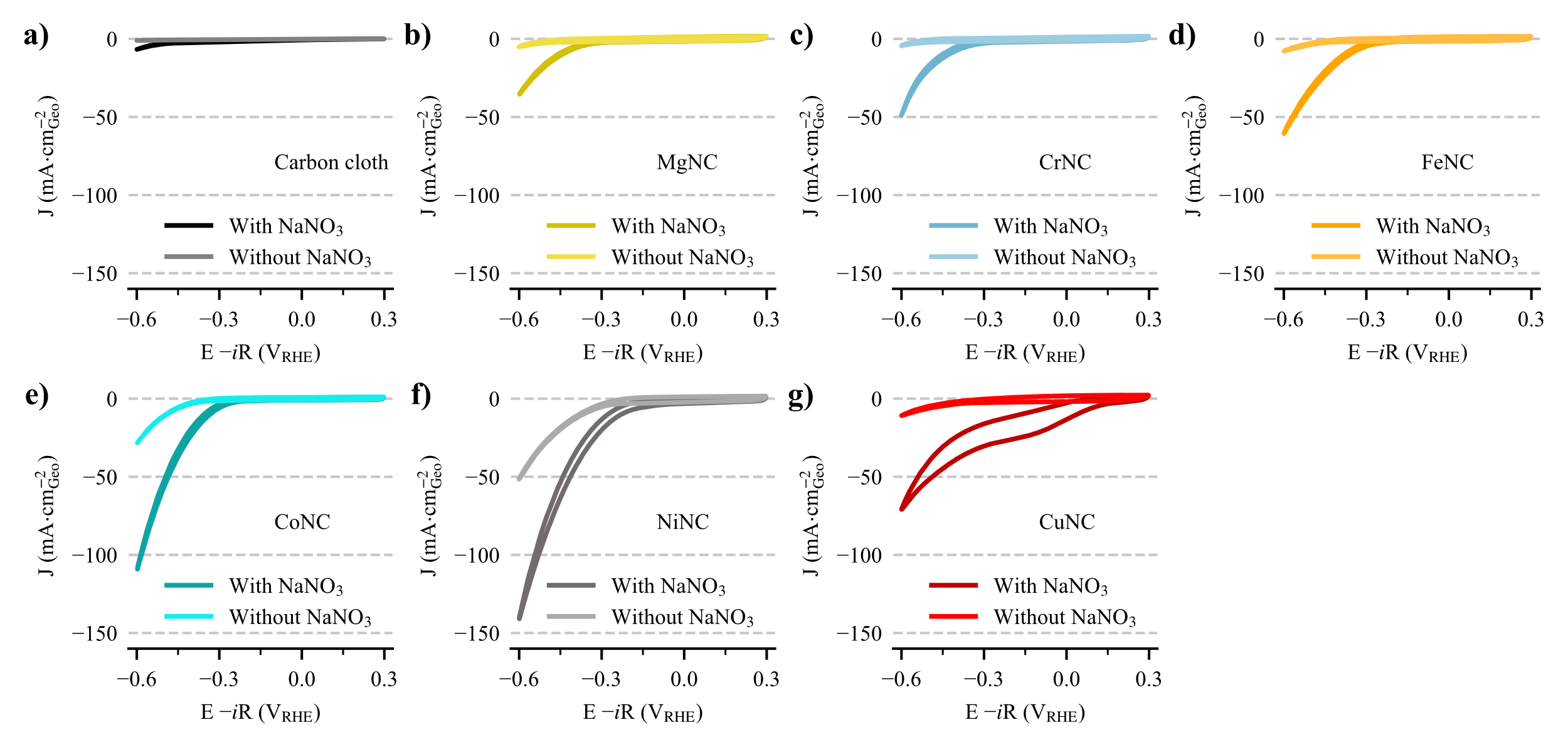


Figure S15 – 5^th^ CV at 20 mV·s^−1^, from +0.3 to −0.6 V_−iR,RHE_, in 0.1 mol·L^−1^ NaOH and in 0.1 mol·L^−1^ NaOH + 0.5 mol·L^−1^ NaNO_3_ with Carbon cloth (a), MgNC (b), CrNC (c), FeNC (d), CoNC (e), NiNC (f) and CuNC (g).


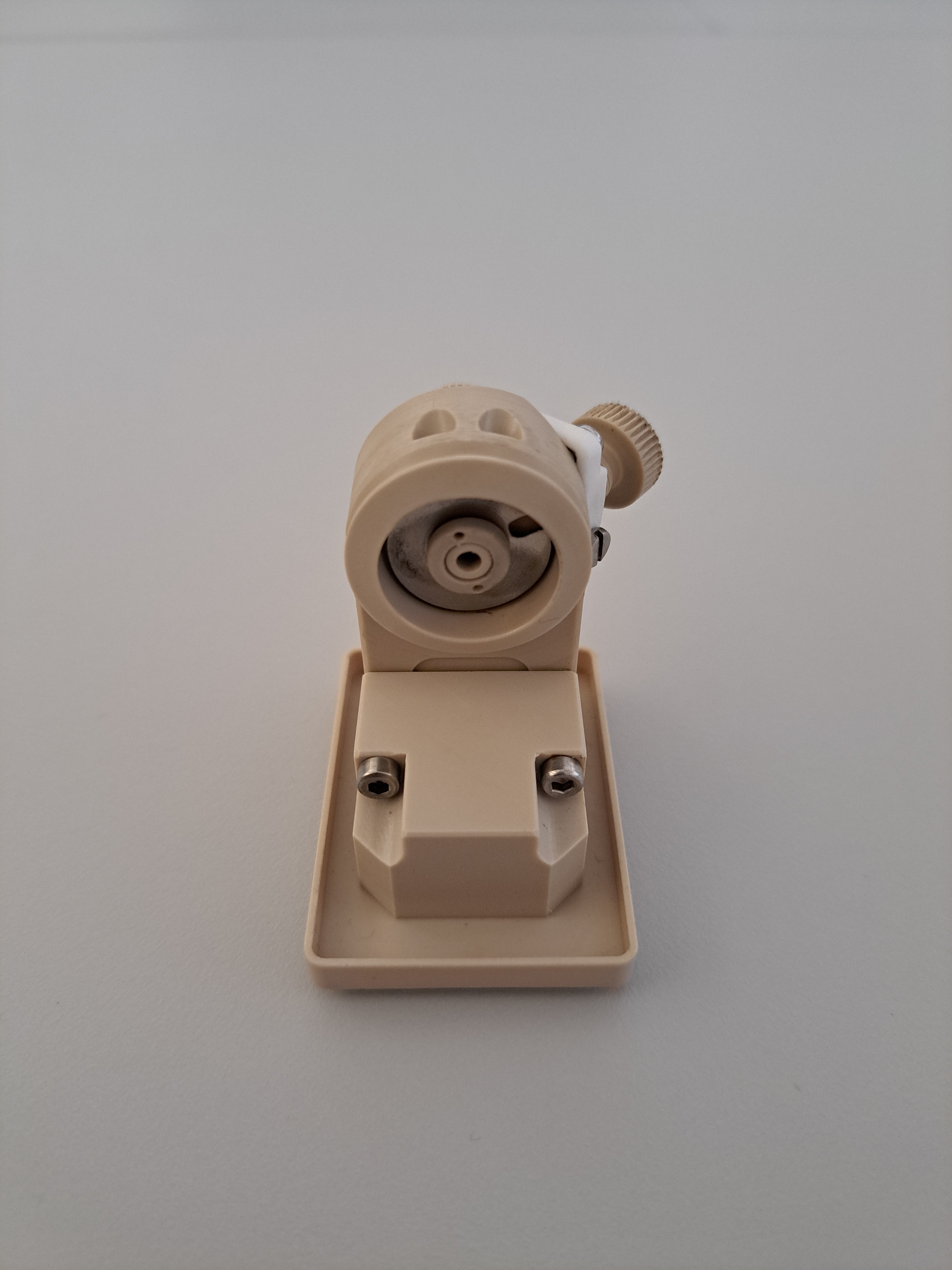


Figure S16 – Photograph of the in situ electrochemical cell used in the Carnaúba synchrotron beamline (LNLS/CNPEM). The working electrode is positioned at the center, the reference electrode is inserted from the right-side inlet, and the counter electrode is the surrounding platinum ring. The front-side is covered by Mylar film. Additional information about the cell can be found in Ref. ^[2,3]^

_
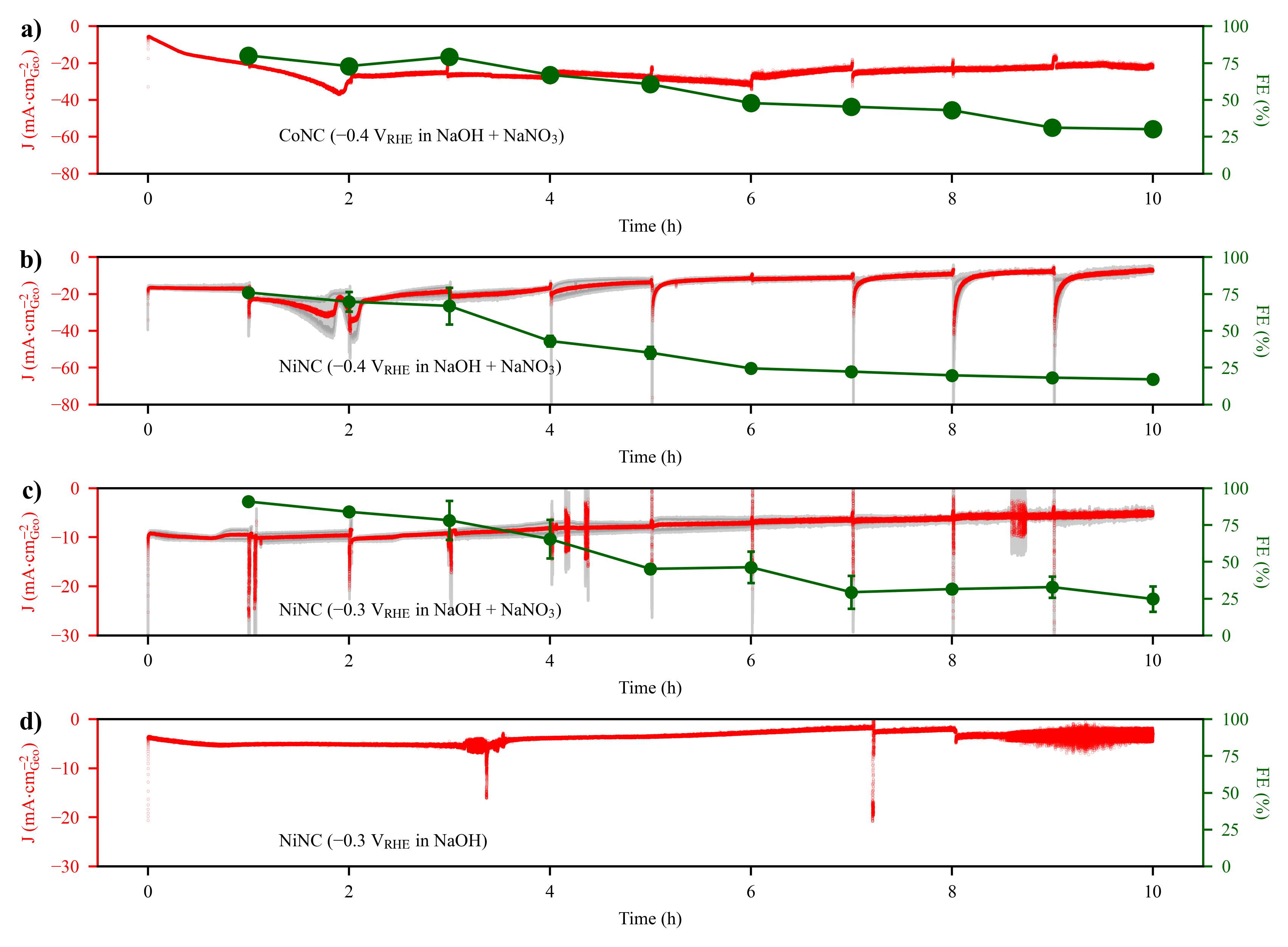
_

Figure S17 – 10-h stability test performed in 0.1 mol·L^−1^ NaOH + 0.5 mol·L^−1^ NaNO_3_ with CoNC at −0.4 V_−iR,RHE_ (a), NiNC at −0.4 V_−iR,RHE_ (b) and −0.3 V_−iR,RHE_ (c), and performed in 0.1 mol·L^−1^ NaOH with NiNC at −0.3 V_−iR,RHE_ (d). FE to ammonia is displayed on right hand y axis. Where the shadows and error bars in (b) and (c) represent the standard deviation of these measures.

**
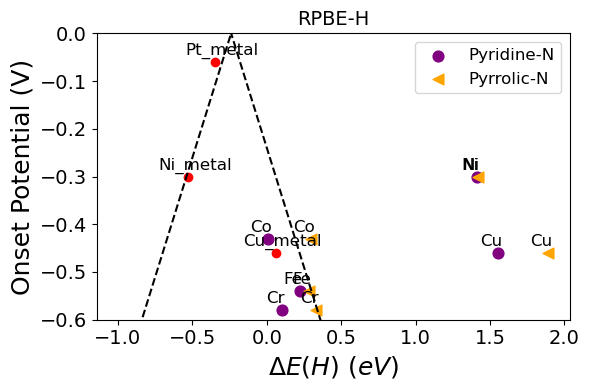
**

*Figure S18* – *HER volcano plot constructed with respect to the onset potential (at 5 mA cm^-2^). The onset potential for Pt(metal) was taken from a previous study.*^[9]^ *The calculated H-adsorption energies were then plotted on the volcano to evaluate their alignment with the predicted activity trends.*


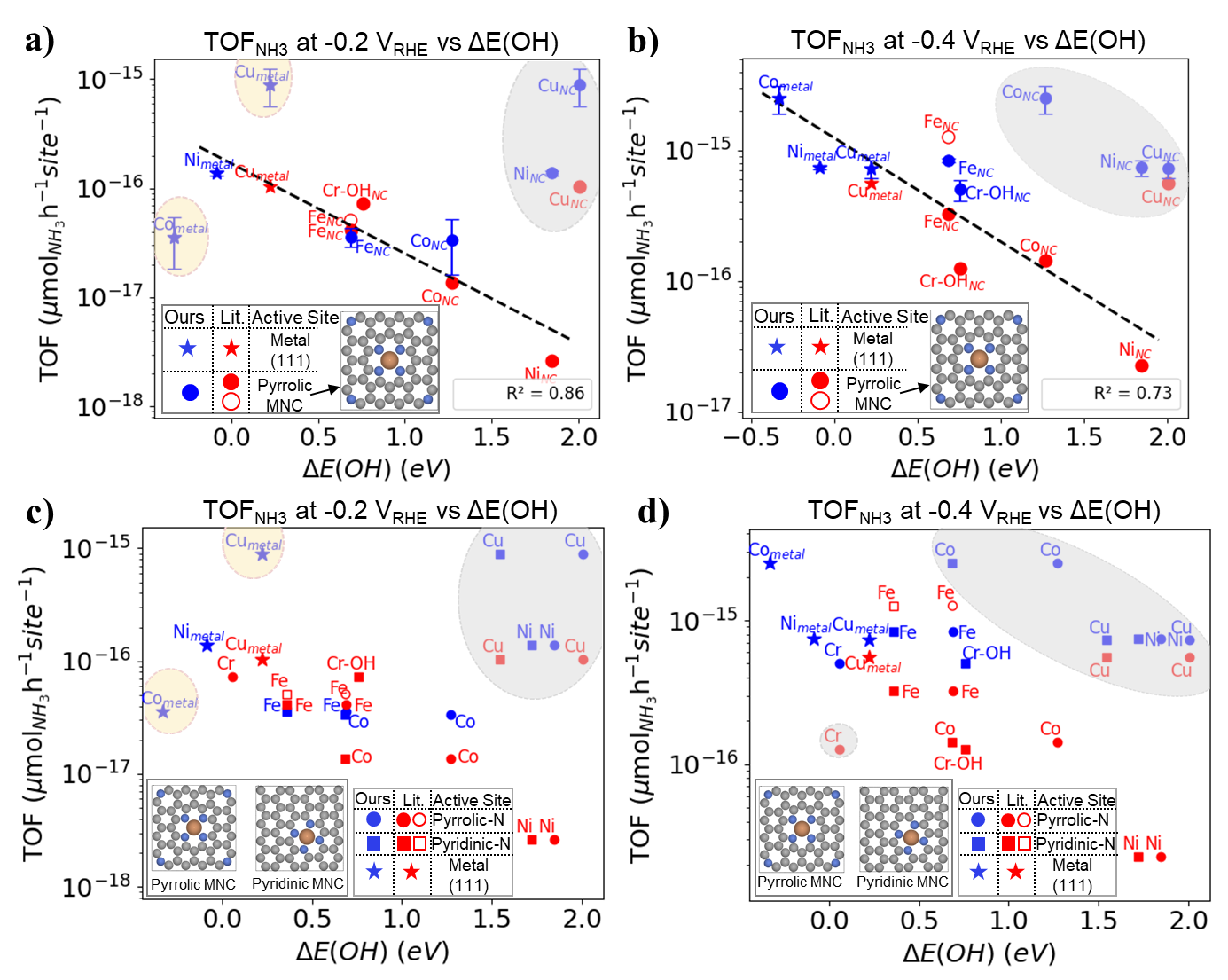


Figure S19 – Correlation between *OH adsorption energy and TOF (µmol_NH3_ h^-1^ site^-1^) for pyrrolic MNC and metal surfaces (FCC (111)) at −0.2 V_RHE_ (a) and −0.4 V_RHE_ (b). Combined pyrrolic and pyridinic MNC and metal surfaces are shown at −0.2 V_RHE_ (c) −0.4 V_RHE_ (d). Our data is shown in blue and TOF calculated from Murphy et al. (0.05 mol L^-1^ PBS + 0.16 mol L^-1^ KNO_3_ electrolyte, 2-h at applied potential) in filled red,^9^ assuming all metal from ICP-MS is coordinated as MNC with 100% electrochemical active site utilization. The hollow symbols for FeNC TOF data are taken from Li et al. (0.1 mol L^-1^ KOH and 0.1 mol L^-1^ KNO_3_ electrolyte, 30 min at applied potential), where TOF was obtained by scanning electrochemical surface interrogation scanning electrochemical microscopy).^11^ The inset shows the pyridinic and pyrrolic computational models used for MNCs. Stars represent metal surfaces for Ni, Co and Cu. The data points in yellow and grey regions were not used in the calculation of R^2^. Error bars correspond to at least two measurements.


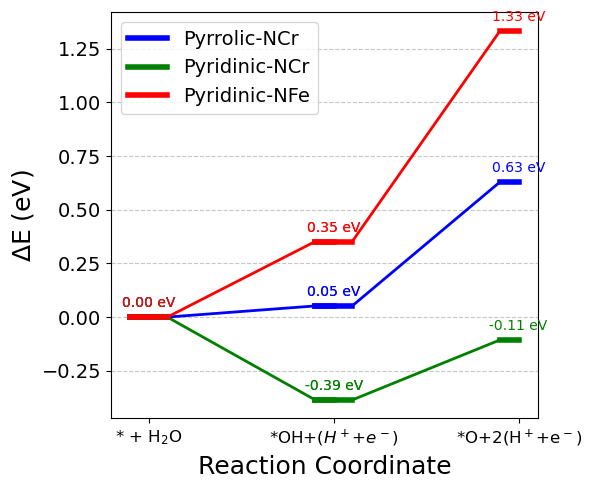


Figure S20 – Energy profile for adsorption of O and OH as back-ligand on the MNCs with reference to free H_2_O at 0 V_RHE_, as defined by the computational hydrogen electrode.

.


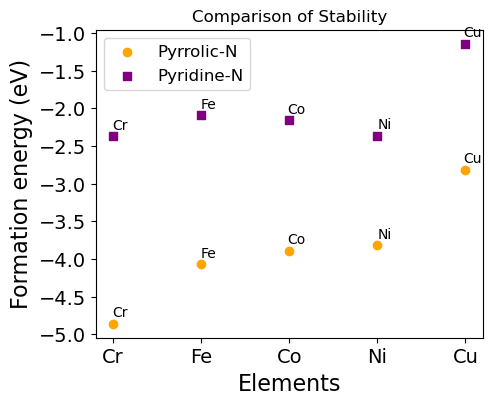


Figure S21 – Formation energy of each MNC calculated with reference to the free NC system and corresponding bulk metal (E_f_ = E_MNC_ –(E_NC_ + 1/n(E_metal_)). Here, n is the number of atoms in the unit cell.


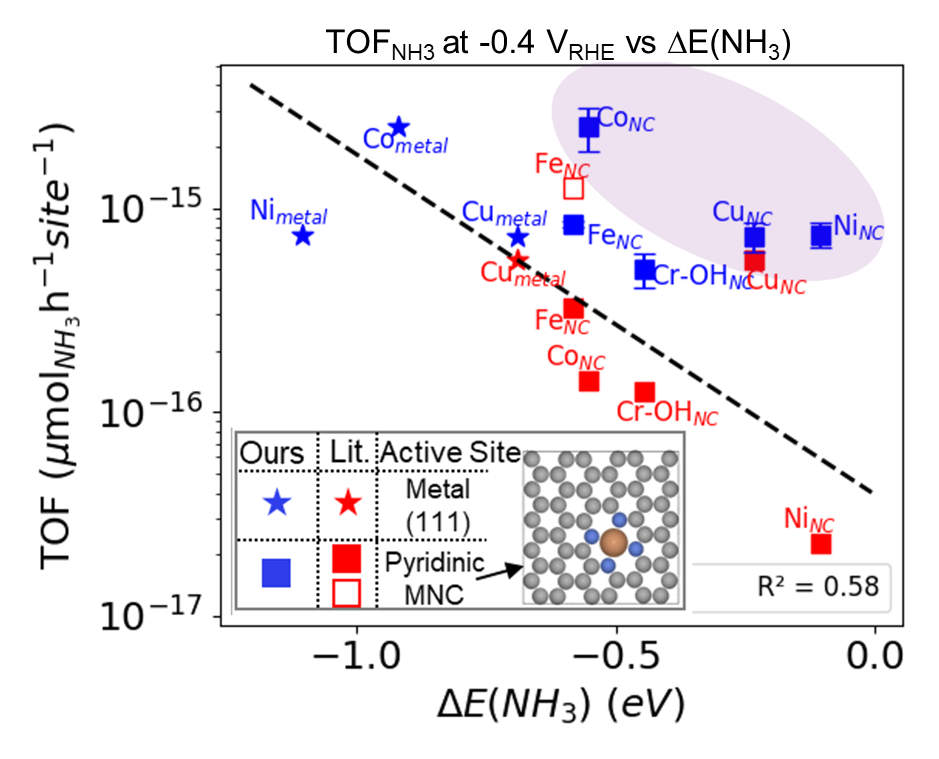


Figure S22 – Correlation between *NH_3_ adsorption energy and TOF (µmol_NH3_ h^-1^ site^-1^) for pyridinic MNC and metal surfaces (FCC (111)) at −0.4 V_RHE_. The inset shows the pyridinic computational models used for MNCs. Stars represent metal surfaces for Ni, Co and Cu. The data points in the grey region were not used in the calculation of R^2^. Error bars correspond to at least two measurements.

*
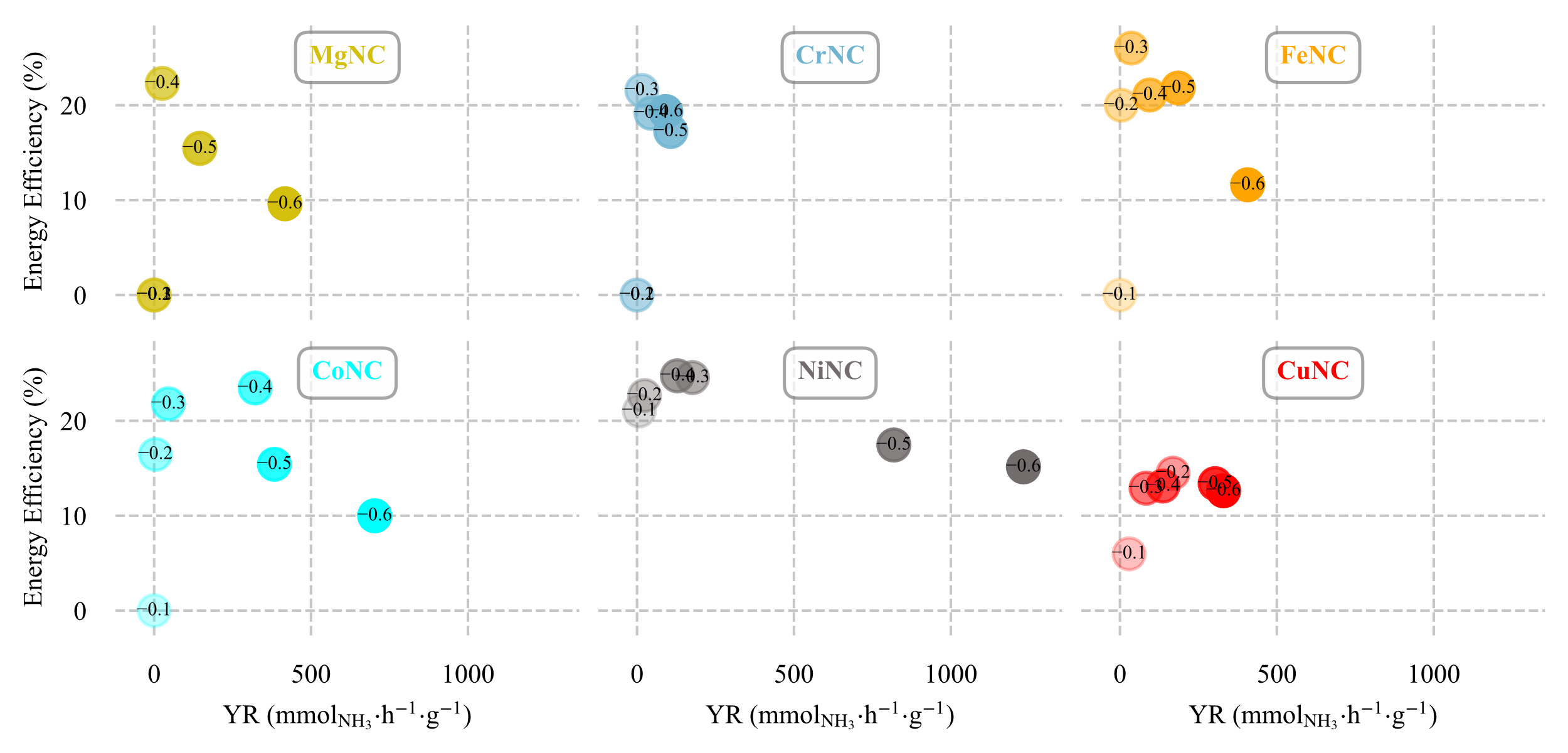
*

Figure S23 – Energy efficiency of all materials investigated in this study. The YR values were normalized by mass of catalyst. Only the mean value of EE and YR are shown.


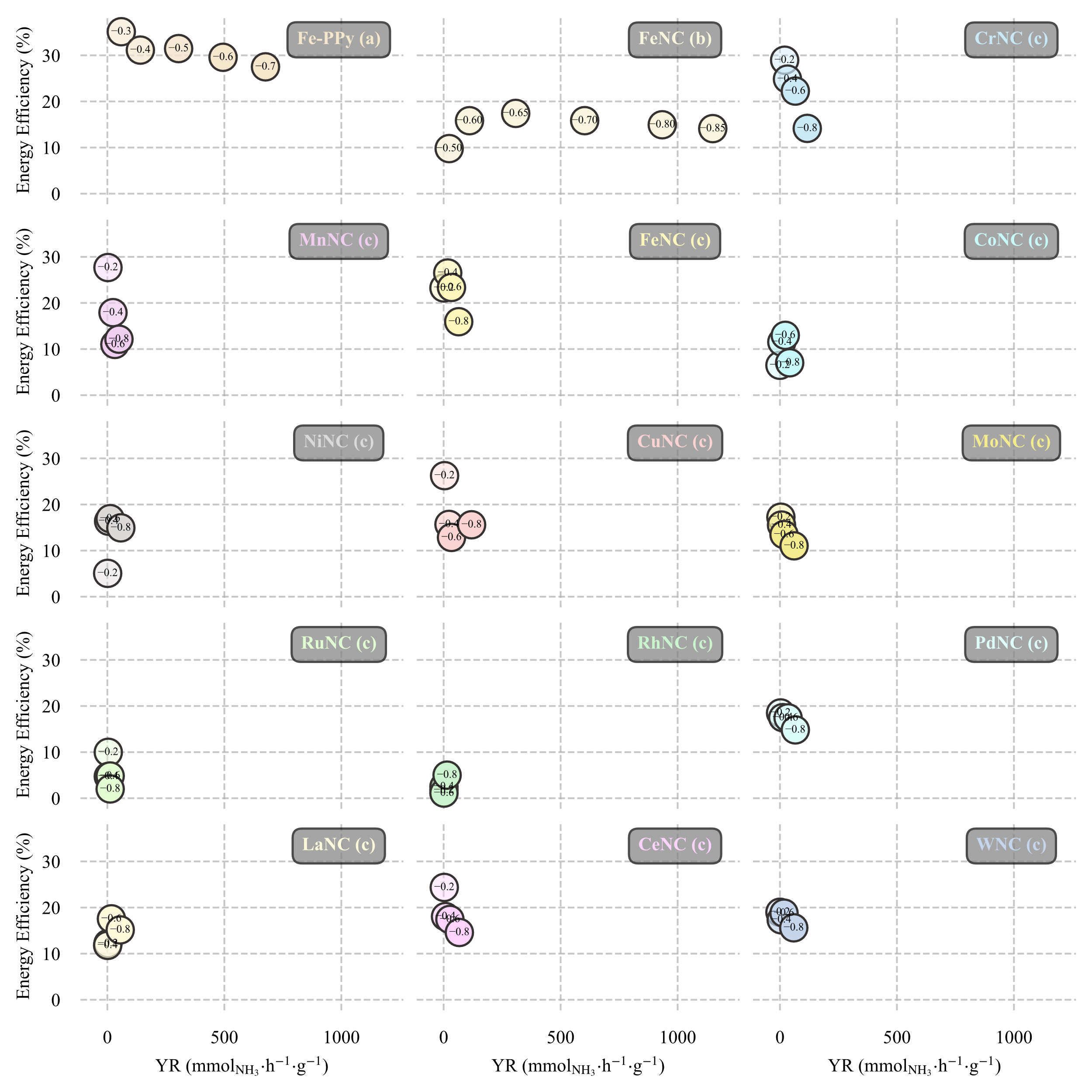


Figure S24 – Energy efficiency of materials in the literature. The YR values were normalized by mass of catalyst. Only the mean value of EE and YR are shown. Ref. a is ^[10]^, Ref. b is ^[11]^ and Ref. c is ^[12]^


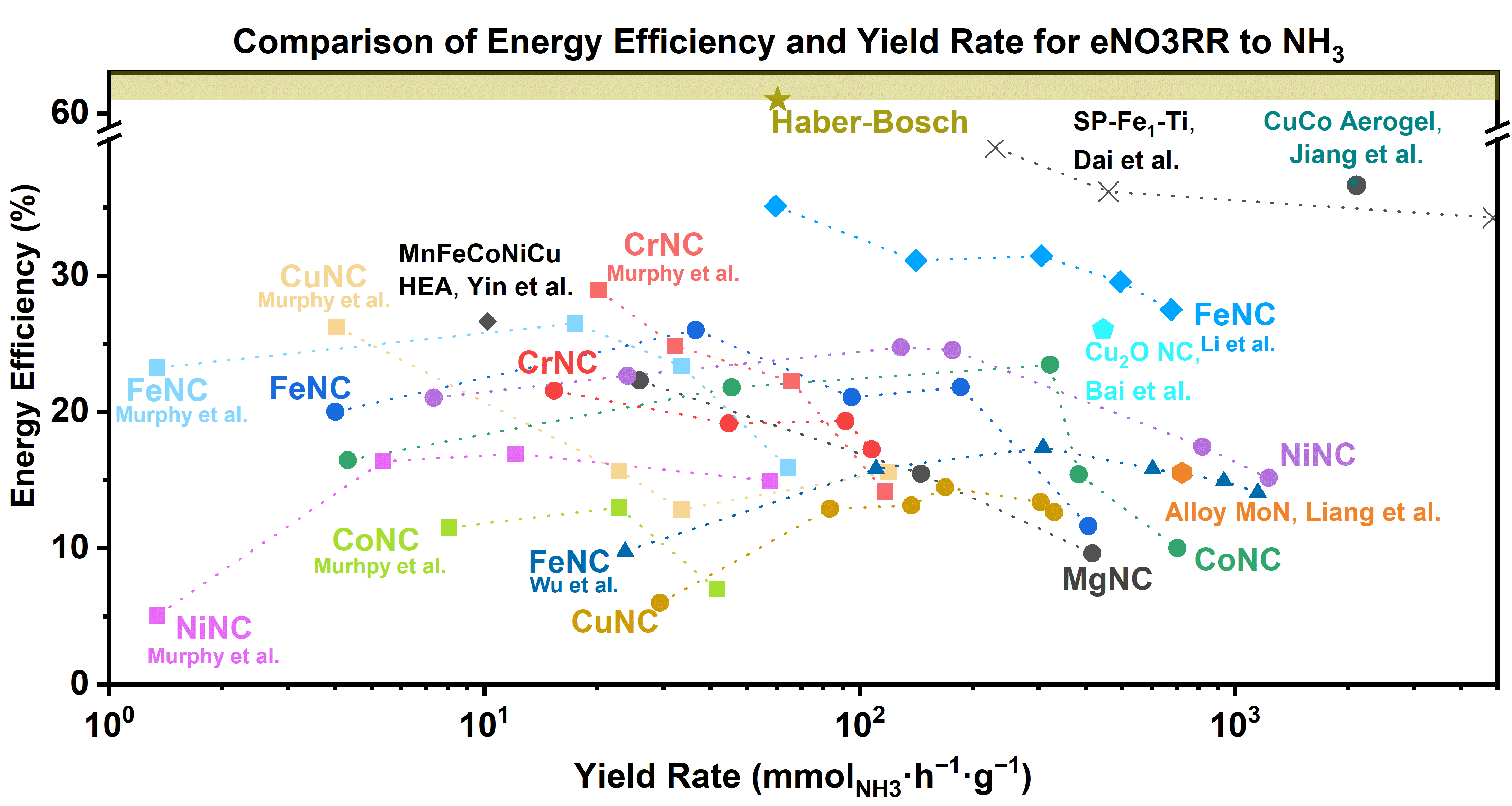


Figure S25 – EE and $\text{YR}_{\text{NH}_{\text{3}}}$ of all MNCs investigated in this study after 1-h electrolysis at the defined potential (0.1 mol·L^−1^ NaOH + 0.5 mol·L^−1^ NaNO_3_ with Ar 99.999% as purging gas in a H-type cell with a graphite bar as CE and a Fumasep membrane to separate the compartments) and literature SACs materials. State-of-the-art non-MNC data: MnFeCuNiCu HEA - Yin et al. (black diamond),^[13]^ SP-Fe_1_-Ti – Dai et al. (black crosses),^[14]^ Cu_2_O NC – Bai et al. (turquoise pentagon),^[15]^ MoN alloy – Liang et al. (orange hexagon),^[16]^ CuCo aerogel (green circle).^[17]^ The YR values here were normalized by mass of catalysts. Only the mean value of EE and YR are being shown here. Individual plot s for each material are provided in Figure S23 and S24 for clarity. Overpotential for counter reaction (oxygen evolution) was assumed to be zero for EE calculation.

Table S2 – Comparison of different materials based on applied potential, yield rate, energy efficiency. Energy efficiency was calculated using the respective pH, the potential is at the respective pH.

| **Material** | **Electrolyte** | **Potential (V)** | **Yield Rate (mmol·h^−1^·g^−1^)** | **Energy Efficiency (%)** | **Ref.** |
| --- | --- | --- | --- | --- | --- |
| MgNC | 0.1 mol·L^−1^ NaOH + 0.5 mol·L^−1^ NaNO_3_ | −0.1 | 0.00 | 0.00 | This work |
|  |  | −0.2 | 0.00 | 0.00 |  |
|  |  | −0.3 | 0.00 | 0.00 |  |
|  |  | −0.4 | 25.88 | 22.30 |  |
|  |  | −0.5 | 145.52 | 15.46 |  |
|  |  | −0.6 | 416.94 | 9.62 |  |
| CrNC | 0.1 mol·L^−1^ NaOH + 0.5 mol·L^−1^ NaNO_3_ | −0.1 | 0.00 | 0.00 | This work |
|  |  | −0.2 | 0.00 | 0.00 |  |
|  |  | −0.3 | 15.34 | 21.56 |  |
|  |  | −0.4 | 44.78 | 19.16 |  |
|  |  | −0.5 | 107.60 | 17.24 |  |
|  |  | −0.6 | 91.68 | 19.33 |  |
| FeNC | 0.1 mol·L^−1^ NaOH + 0.5 mol·L^−1^ NaNO_3_ | −0.1 | 0.00 | 0.00 | This work |
|  |  | −0.2 | 4.00 | 20.00 |  |
|  |  | −0.3 | 36.54 | 26.02 |  |
|  |  | −0.4 | 95.20 | 21.08 |  |
|  |  | −0.5 | 185.72 | 21.81 |  |
|  |  | −0.6 | 406.98 | 11.62 |  |
| CoNC | 0.1 mol·L^−1^ NaOH + 0.5 mol·L^−1^ NaNO_3_ | −0.1 | 0.00 | 0.00 | This work |
|  |  | −0.2 | 4.32 | 16.46 |  |
|  |  | −0.3 | 45.54 | 21.79 |  |
|  |  | −0.4 | 321.78 | 23.46 |  |
|  |  | −0.5 | 383.16 | 15.42 |  |
|  |  | −0.6 | 702.78 | 9.99 |  |
| NiNC | 0.1 mol·L^−1^ NaOH + 0.5 mol·L^−1^ NaNO_3_ | −0.1 | 7.32 | 21.04 | This work |
|  |  | −0.2 | 24.04 | 22.66 |  |
|  |  | −0.3 | 176.52 | 24.55 |  |
|  |  | −0.4 | 128.72 | 24.74 |  |
|  |  | −0.5 | 818.34 | 17.45 |  |
|  |  | −0.6 | 1231.40 | 15.15 |  |
| CuNC | 0.1 mol·L^−1^ NaOH + 0.5 mol·L^−1^ NaNO_3_ | −0.1 | 29.38 | 5.98 | This work |
|  |  | −0.2 | 169.08 | 14.48 |  |
|  |  | −0.3 | 83.24 | 12.90 |  |
|  |  | −0.4 | 137.12 | 13.13 |  |
|  |  | −0.5 | 303.40 | 13.38 |  |
|  |  | −0.6 | 330.04 | 12.63 |  |
| Fe-PPy | 0.1 mol·L^−1^ KOH + 0.1 mol·L^−1^ KNO_3_ | −0.3 | 59.67 | 35.12 | ^[10]^ |
|  |  | −0.4 | 141.13 | 31.13 |  |
|  |  | −0.5 | 304.63 | 31.46 |  |
|  |  | −0.6 | 494.58 | 29.57 |  |
|  |  | −0.7 | 675.92 | 27.50 |  |
| CrNC | 0.05 mol·L^−1^ PBS + 0.16 mol·L^−1^ KNO_3_ | −0.2 | 20.12 | 28.94 | ^[12]^ |
|  |  | −0.4 | 32.20 | 24.84 |  |
|  |  | −0.6 | 65.76 | 22.24 |  |
|  |  | −0.8 | 116.76 | 14.15 |  |
| MnNC | 0.05 mol·L^−1^ PBS + 0.16 mol·L^−1^ KNO_3_ | −0.2 | 2.68 | 27.68 | ^[12]^ |
|  |  | −0.4 | 24.16 | 17.90 |  |
|  |  | −0.6 | 32.20 | 11.00 |  |
|  |  | −0.8 | 49.66 | 12.14 |  |
| FeNC | 0.05 mol·L^−1^ PBS + 0.16 mol·L^−1^ KNO_3_ | −0.2 | 1.34 | 23.25 | ^[12]^ |
|  |  | −0.4 | 17.44 | 26.50 |  |
|  |  | −0.6 | 33.54 | 23.36 |  |
|  |  | −0.8 | 64.42 | 15.93 |  |
| CoNC | 0.05 mol·L^−1^ PBS + 0.16 mol·L^−1^ KNO_3_ | −0.2 | 0.00 | 6.48 | ^[12]^ |
|  |  | −0.4 | 8.04 | 11.52 |  |
|  |  | −0.6 | 22.80 | 12.98 |  |
|  |  | −0.8 | 41.60 | 7.02 |  |
| NiNC | 0.05 mol·L^−1^ PBS + 0.16 mol·L^−1^ KNO_3_ | −0.2 | 1.34 | 5.06 | ^[12]^ |
|  |  | −0.4 | 5.36 | 16.37 |  |
|  |  | −0.6 | 12.08 | 16.93 |  |
|  |  | −0.8 | 57.70 | 14.93 |  |
| CuNC | 0.05 mol·L^−1^ PBS + 0.16 mol·L^−1^ KNO_3_ | −0.2 | 4.03 | 26.25 | ^[12]^ |
|  |  | −0.4 | 22.80 | 15.68 |  |
|  |  | −0.6 | 33.54 | 12.85 |  |
|  |  | −0.8 | 119.46 | 15.60 |  |
| MoNC | 0.05 mol·L^−1^ PBS + 0.16 mol·L^−1^ KNO_3_ | −0.2 | 4.02 | 17.24 | ^[12]^ |
|  |  | −0.4 | 6.70 | 15.54 |  |
|  |  | −0.6 | 17.44 | 13.47 |  |
|  |  | −0.8 | 60.40 | 11.03 |  |
| RuNC | 0.05 mol·L^−1^ PBS + 0.16 mol·L^−1^ KNO_3_ | −0.2 | 4.03 | 9.96 | ^[12]^ |
|  |  | −0.4 | 4.03 | 4.72 |  |
|  |  | −0.6 | 12.08 | 4.82 |  |
|  |  | −0.8 | 12.08 | 2.01 |  |
| RhNC | 0.05 mol·L^−1^ PBS + 0.16 mol·L^−1^ KNO_3_ | −0.2 | 1.34 | 1.74 | ^[12]^ |
|  |  | −0.4 | 1.34 | 2.50 |  |
|  |  | −0.6 | 1.34 | 1.11 |  |
|  |  | −0.8 | 14.76 | 5.01 |  |
| PdNC | 0.05 mol·L^−1^ PBS + 0.16 mol·L^−1^ KNO_3_ | −0.2 | 2.68 | 18.50 | ^[12]^ |
|  |  | −0.4 | 12.08 | 17.48 |  |
|  |  | −0.6 | 34.88 | 17.42 |  |
|  |  | −0.8 | 65.76 | 14.82 |  |
| LaNC | 0.05 mol·L^−1^ PBS + 0.16 mol·L^−1^ KNO_3_ | −0.2 | 2.68 | 12.18 | ^[12]^ |
|  |  | −0.4 | 1.34 | 11.79 |  |
|  |  | −0.6 | 17.44 | 17.55 |  |
|  |  | −0.8 | 55.02 | 15.15 |  |
| CeNC | 0.05 mol·L^−1^ PBS + 0.16 mol·L^−1^ KNO_3_ | −0.2 | 2.68 | 24.36 | ^[12]^ |
|  |  | −0.4 | 8.04 | 18.04 |  |
|  |  | −0.6 | 26.84 | 17.42 |  |
|  |  | −0.8 | 67.10 | 14.59 |  |
| WNC | 0.05 mol·L^−1^ PBS + 0.16 mol·L^−1^ KNO_3_ | −0.2 | 0.00 | 18.98 | ^[12]^ |
|  |  | −0.4 | 5.36 | 17.34 |  |
|  |  | −0.6 | 17.44 | 18.78 |  |
|  |  | −0.8 | 59.06 | 15.60 |  |
| FeNC | 0.50  mol·L^−1^ KNO_3_ + 0.10  mol·L^−1^ K_2_SO_4_ | −0.50 | 23.69 | 9.77 | ^[11]^ |
|  |  | −0.60 | 110.54 | 15.82 |  |
|  |  | −0.65 | 307.94 | 17.40 |  |
|  |  | −0.70 | 602.71 | 15.83 |  |
|  |  | −0.80 | 934.33 | 14.94 |  |
|  |  | −0.85 | 1150.15 | 14.10 |  |
| MnFeCoNiCu HEA | 0.5 mol·L^−1^ Na_2_SO_4_ + 0.1 mol·L^−1^ NO_3_^−^ | −0.60 | 10.2 | 26.65 | ^[13]^ |
| SP-Fe_1_-Ti | 1.0 mol·L^−1^ KOH + 1.0 mol·L^−1^ KNO_3_ | −0.1 | 230 | 39.40 | ^[14]^ |
|  |  | −0.2 | 460 | 36.17 |  |
|  |  | −0.3 | 4870 | 34.25 |  |
|  |  | −0.4 | 15770 | 30.58 |  |
|  |  | −0.5 | 19940 | 25.86 |  |
|  |  | −0.6 | 32460 | 23.57 |  |
|  |  | −0.7 | 38960 | 21.64 |  |
| Cu_2_O NCs | 0.1 mol·L^−1^ NaSO_4_ + 8 mmol·L^−1^ NaNO_3_ | −0.3 | 445.29 | 26.07 | ^[15]^ |
| Alloy-MoN | 0.1 mol·L^−1^ K_2_SO_4_ and 0.1 mol·L^−1^ KNO_3_ | −1.2 | 722.35 | 15.53 | ^[16]^ |
| CuCo aerogel | 1.0 mol·L^−1^ KOH + KNO_3_ 0.07 mol·L^−1^ | −0.2 | 2110 | 36.64 | ^[17]^ |
| Ru | H-B process | - | 60.40 | 61 | ^[10,18]^ |

^*The values from references 11-17 were extracted from the images.^

**References**

[1] A. Mehmood, M. Gong, F. Jaouen, A. Roy, A. Zitolo, A. Khan, M.-T. Sougrati, M. Primbs, A. M. Bonastre, D. Fongalland, G. Drazic, P. Strasser, A. Kucernak, **High loading of single atomic iron sites in Fe–NC oxygen reduction catalysts for proton exchange membrane fuel cells** *Nat Catal* **2022**, *5*, 311–323.

[2] G. C. Sedenho, I. T. Neckel, R. N. P. Colombo, J. C. Pacheco, T. Bertaglia, F. N. Crespilho, **Investigation of Water Splitting Reaction by a Multicopper Oxidase through X‐ray Absorption Nanospectroelectrochemistry** *Advanced Energy Materials* **2022**, *12*, 2202485.

[3] R. A. Vicente, I. T. Neckel, P. S. Fernández, **Mastering the Use of Nanoprobes Beamlines for Electrochemistry: The Importance of Tracking Radiation Damage and Exploring Material Heterogeneity** *ACS Electrochem.* **2025**, *1*, 286–293.

[4] P. E. Blöchl, **Projector augmented-wave method** *Phys. Rev. B* **1994**, *50*, 17953–17979.

[5] G. Kresse, J. Furthmüller, **Efficient iterative schemes for *ab initio* total-energy calculations using a plane-wave basis set** *Phys. Rev. B* **1996**, *54*, 11169–11186.

[6] B. Hammer, L. B. Hansen, J. K. Nørskov, **Improved adsorption energetics within density-functional theory using revised Perdew-Burke-Ernzerhof functionals** *Phys. Rev. B* **1999**, *59*, 7413–7421.

[7] S. Grimme, J. Antony, S. Ehrlich, H. Krieg, **A consistent and accurate *ab initio* parametrization of density functional dispersion correction (DFT-D) for the 94 elements H-Pu** *The Journal of Chemical Physics* **2010**, *132*, 154104.

[8] J. K. Nørskov, T. Bligaard, A. Logadottir, J. R. Kitchin, J. G. Chen, S. Pandelov, U. Stimming, **Trends in the Exchange Current for Hydrogen Evolution** *J. Electrochem. Soc.* **2005**, *152*, J23.

[9] A. N. Nair, M. F. Sanad, V. S. N. Chava, S. T. Sreenivasan, **Platinum-like HER onset in a GNR/MoS_2_ quantum dot heterostructure through curvature-dependent electron density reconfiguration** *Chem. Commun.* **2022**, *58*, 10368–10371.

[10] P. Li, Z. Jin, Z. Fang, G. Yu, **A single-site iron catalyst with preoccupied active centers that achieves selective ammonia electrosynthesis from nitrate** *Energy Environ. Sci.* **2021**, *14*, 3522–3531.

[11] Z.-Y. Wu, M. Karamad, X. Yong, Q. Huang, D. A. Cullen, P. Zhu, C. Xia, Q. Xiao, M. Shakouri, F.-Y. Chen, J. Y. (Timothy) Kim, Y. Xia, K. Heck, Y. Hu, M. S. Wong, Q. Li, I. Gates, S. Siahrostami, H. Wang, **Electrochemical ammonia synthesis via nitrate reduction on Fe single atom catalyst** *Nat Commun* **2021**, *12*, 2870.

[12] E. Murphy, Y. Liu, I. Matanovic, M. Rüscher, Y. Huang, A. Ly, S. Guo, W. Zang, X. Yan, A. Martini, J. Timoshenko, B. R. Cuenya, I. V. Zenyuk, X. Pan, E. D. Spoerke, P. Atanassov, **Elucidating electrochemical nitrate and nitrite reduction over atomically-dispersed transition metal sites** *Nat Commun* **2023**, *14*, 4554.

[13] D. Yin, B. Li, B. Gao, M. Chen, D. Chen, Y. Meng, S. Zhang, C. Zhang, Q. Quan, L. Chen, C. Yang, C. Wong, J. C. Y. Ho, **Overcoming Energy‐Scaling Barriers: Efficient Ammonia Electrosynthesis on High‐Entropy Alloy Catalysts** *Advanced Materials* **2025**, *37*, DOI 10.1002/adma.202415739.

[14] J. Dai, Y. Tong, L. Zhao, Z. Hu, C.-T. Chen, C.-Y. Kuo, G. Zhan, J. Wang, X. Zou, Q. Zheng, W. Hou, R. Wang, K. Wang, R. Zhao, X.-K. Gu, Y. Yao, L. Zhang, **Spin polarized Fe1−Ti pairs for highly efficient electroreduction nitrate to ammonia** *Nat Commun* **2024**, *15*, DOI 10.1038/s41467-023-44469-4.

[15] L. Bai, F. Franco, J. Timoshenko, C. Rettenmaier, F. Scholten, H. S. Jeon, A. Yoon, M. Rüscher, A. Herzog, F. T. Haase, S. Kühl, S. W. Chee, A. Bergmann, R. C. Beatriz, **Electrocatalytic Nitrate and Nitrite Reduction toward Ammonia Using Cu_2_O Nanocubes: Active Species and Reaction Mechanisms** *J. Am. Chem. Soc.* **2024**, *146*, 9665–9678.

[16] H. Liang, M. Chen, Y. Feng, G. Meng, J. Zhang, W. Liu, X. Liu, **Construction of a Heterostructured Alloy–Molybdenum Nitride Catalyst for Enhanced NH_3_ Production via Nitrate Electrolysis** *Inorg. Chem.* **2025**, *64*, 1252–1257.

[17] Z. Jiang, S. Jiang, W. Huang, S. Li, S. Chen, H. Li, G. Zheng, J. Yang, **High‐Performance CuCo Aerogel Electrocatalyst for Relay Electroreduction of Nitrate to Ammonia** *Adv Funct Materials* **2025**, DOI 10.1002/adfm.202507903.

[18] M. Kitano, Y. Inoue, M. Sasase, K. Kishida, Y. Kobayashi, K. Nishiyama, T. Tada, S. Kawamura, T. Yokoyama, M. Hara, H. Hosono, **Self‐organized Ruthenium–Barium Core–Shell Nanoparticles on a Mesoporous Calcium Amide Matrix for Efficient Low‐Temperature Ammonia Synthesis** *Angew Chem Int Ed* **2018**, *57*, 2648–2652.
